# Cardioneuroablation for Vasovagal Syncope: An Updated Systematic Review and Single-Arm Meta-Analysis

**DOI:** 10.3390/biomedicines13071758

**Published:** 2025-07-18

**Authors:** Alexandru Ababei, Cosmin Gabriel Ursu, Mircea Ioan Alexandru Bistriceanu, Darie Ioan Andreescu, Iasmina-Maria Iurea, Beatrice Budeanu, Adriana Elena Dumitrache, Alexandra Hostiuc, Maria-Celina Sturz-Lazar, Cristian-Valentin Toma, Stefan Sebastian Busnatu, Alexandru Deaconu, Stefan Bogdan

**Affiliations:** 1 Iliescu Emergency Institute for Cardiovascular Diseases, 022328 Bucharest, Romania; alexandru.ababei0125@rez.umfcd.ro; 2 Faculty of Medicine, Carol Davila University of Medicine and Pharmacy, 050474 Bucharest, Romania; cosmin.d.v.ursu@stud.umfcd.ro (C.G.U.); darie-ioan.andreescu0720@stud.umfcd.ro (D.I.A.); iasmina-maria.iurea0720@stud.umfcd.ro (I.-M.I.); beatrice.budeanu0720@stud.umfcd.ro (B.B.); adriana-elena.dumitrache0720@stud.umfcd.ro (A.E.D.); alexandra.hostiuc0920@stud.umfcd.ro (A.H.); maria-celina.falota0720@stud.umfcd.ro (M.-C.S.-L.); cristian.toma@umfcd.ro (C.-V.T.); stefan.busnatu@umfcd.ro (S.S.B.); alexandru.deaconu@umfcd.ro (A.D.); stefan.bogdan@umfcd.ro (S.B.); 3 Bagdasar-Arseni Emergency Hospital, 041915 Bucharest, Romania; 4 Department of Cardio-Thoracic Pathology, Faculty of Medicine, “Carol Davila” University of Medicine and Pharmacy, 050474 Bucharest, Romania; 5 Emergency Clinical Hospital, 014461 Bucharest, Romania; 6 Elias Clinical Emergency Hospital, 011461 Bucharest, Romania

**Keywords:** vasovagal syncope, cardioneuroablation, ganglionated plexus

## Abstract

**Background:** When conservative therapies are insufficient for vasovagal syncope (VVS), procedural options such as permanent pacemakers or catheter ablation of ganglionated plexi (GP) may be considered. This meta-analysis aimed to evaluate the efficacy of GP catheter ablation in patients with VVS. **Methods:** A comprehensive literature search was performed in PubMed, Embase, and the Cochrane Library from 15 March 2024 to 10 May 2025. After duplicate removal, two reviewers independently screened studies and assessed full texts based on predefined criteria. A single-arm proportion meta-analysis was conducted. **Results:** Thirty-seven studies comprising 1585 participants were included. The pooled proportion of VVS recurrence after ablation was 8.9% (95% CI, 6.4–11.4%), but with substantial heterogeneity (I^2^ = 74.4%, *p* < 0.001). Sensitivity and subgroup analyses confirmed the robustness of the pooled estimate. A meta-regression was performed to further explore potential effect modifiers, but no covariate reached statistical significance. **Conclusions:** This meta-analysis suggests that ganglionated plexi catheter ablation may be associated with a reduced recurrence of vasovagal syncope in selected populations. However, the findings are based predominantly on non-randomized observational studies, and the high between-study heterogeneity limits the strength of inference. Future randomized controlled trials with standardized methodologies are needed to confirm the long-term efficacy and safety of this intervention.

## 1. Introduction

Vasovagal syncope (VVS) is the most common form of reflex syncope, often triggered by emotional stress or orthostatic posture without identifiable structural heart disease [1,2]. It affects between 20% and 40% of the general population, with a bimodal age distribution, peaking in adolescence and again after the age of 60 [3,4]. Women are more frequently affected than men. Although VVS is generally considered benign in terms of mortality, it can lead to recurrent episodes, reduced quality of life, and traumatic injuries [5,6]. A recent cohort study reported that approximately 23% of patients with VVS experienced injury during syncopal events [7]. These events also contribute to substantial healthcare utilization and anxiety for both patients and providers. First-line management typically involves patient education and non-pharmacological interventions [2]. When these fail, pharmacological agents, such as midodrine, etilefrine, fluoxetine, and atomoxetine, may be considered [8,9,10,11]. Among these, midodrine has shown the most consistent benefit, though overall pharmacological effectiveness remains limited [12]. In refractory cases, procedural options, such as permanent pacemaker implantation or cardioneuroablation (CNA), have emerged. CNA aims to selectively denervate parasympathetic input by the ablation of atrial ganglionated plexi (AGP), thereby reducing vagal tone [13,14,15,16]. However, most available evidence for CNA is observational, and significant uncertainty remains regarding its safety, efficacy, and optimal procedural endpoints.

Therefore, we conducted an updated systematic review and single-arm meta-analysis to evaluate the clinical impact of CNA in patients with vasovagal syncope. We hypothesized that CNA is associated with a reduction in the recurrence rate of syncope compared to baseline, as well as measurable changes in autonomic parameters, such as heart rate and SDNN.

## 2. Materials and Methods

**Figure 1:**
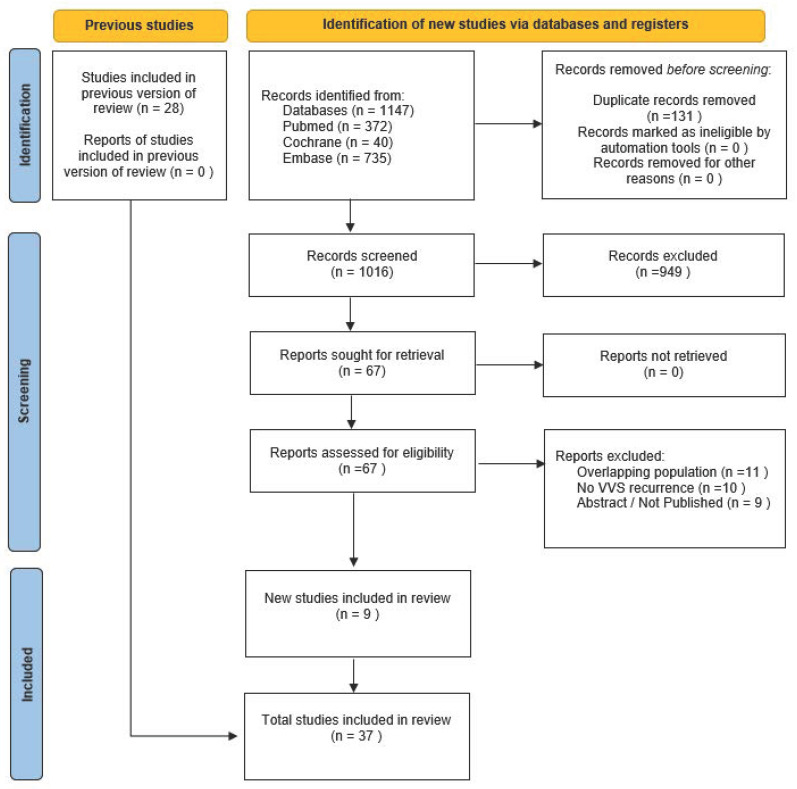
Flow diagram for material preparation and methodological framework of this study [17].

This systematic review and meta-analysis were conducted and reported in accordance with the Cochrane Collaboration Handbook for Systematic Review of Interventions and the Preferred Reporting Items for Systematic Reviews and Meta-Analysis (PRISMA) statement guidelines (Figure 1) [17].

Inclusion in this meta-analysis was restricted to studies that met all of the following eligibility criteria: (1) randomized clinical trials or observational studies; (2) evaluated the recurrence of vasovagal syncope; (3) enrolled patients who underwent cardioneuroablation; and (4) had a follow-up of at least 6 months. Additionally, studies were included only if they reported any of the clinical outcomes of interest. A minimum follow-up of 6 months was chosen based on a preliminary literature review, which found substantial heterogeneity in follow-up durations between different studies. We excluded studies with (1) interventions on animals, (2) less than 5 months of follow-up, (3) no reported vasovagal syncope recurrence, (4) patients with orthostatic hypotension, (5) patients with postural orthostatic tachycardia syndrome (POTS), and (6) other types of syncope.

We systematically searched PubMed, Embase, and the Cochrane Central Register of Controlled Trials from inception to April 2025 using the following search strategy: (syncope OR “vagal reaction” OR “vagal episode”) AND (cardioneuroablation OR “Ganglionated plexus ablation” OR “Neurocardiac Ablation” OR “ Denervation”). The references of all included studies, previous systematic reviews, and meta-analyses were also manually searched for additional studies. Two authors (A.A. and C.U.) independently extracted the data according to predefined search criteria and quality assessment. The prospective meta-analysis protocol was registered on PROSPERO on 25 April 2025, under protocol #CRD420251026927.

Our objective was to analyse (1) the recurrence of syncopal episodes as the primary endpoint for assessing the success of the intervention, alongside the following secondary endpoints: (2) the post-ablation heart rate; (3) the rate of reintervention; (4) the standard deviation of the normal-to-normal sinus node-initiated R-R intervals (SDNN) pre-ablation; and (5) the standard deviation of the normal-to-normal sinus node-initiated R-R intervals (SDNN) post-ablation. Prespecified subanalyses included data limited to (1) RCTs; (2) types of energy used in thermic ablation; (3) the location of CNA; (4) the method of ablation, and (5) age.

We assessed the risk of bias using the Newcastle–Ottawa assessment tool. Two independent authors carried out the risk of bias assessment (A.A. and D.A.). Any disagreements were resolved through consensus after discussing the reasons for the discrepancy.

The meta-analysis employed a single-arm proportion model. The endpoints were calculated as proportions and reported with 95% confidence intervals (CIs). Continuous outcomes were evaluated using standard mean differences.

A random-effects model was chosen for this analysis to account for both within-study sampling errors and between-study heterogeneity. This approach is the recommended method by Cochrane due to the variability of study designs being so prevalent that it is nearly impossible to have true, identically methodological studies. Between-study variance (τ^2^) was estimated using the DerSimonian–Laird method, which remains a standard approach in random-effects meta-analyses for its interpretability and robust performance across varying levels of heterogeneity. Heterogeneity was assessed using τ^2^, I^2^ (percentage of total variability attributable to heterogeneity), and H^2^ (ratio of total to within-study variability). In addition, Cochran’s Q test was applied to statistically evaluate the presence of heterogeneity. Because the Q test has limited power with smaller sample sizes, a significance level of 0.10 was used instead of the conventional 0.05, increasing sensitivity to detect true heterogeneity.

Due to the binomial structure of the data and the inclusion of studies with zero events, the arcsine transformation (PAS method) was applied to stabilize variances across the full range of proportions, including rare events. This transformation mitigates the disproportionate influence of studies with very high or very low event rates on the overall pooled estimate. To further accommodate studies reporting zero recurrences, a continuity correction of 0.5 was applied selectively to those studies, allowing their inclusion without artificially inflating variance estimates.

To assess the contribution of individual studies to the overall heterogeneity and their influence on the pooled effect size, a Baujat plot was generated using the meta package in R. This diagnostic tool plots each study according to its contribution to the total heterogeneity (x-axis) and its influence on the overall meta-analytic result (y-axis).

To explore potential sources of between-study heterogeneity in recurrence rates, a mixed-effects meta-regression was conducted using arcsine-transformed proportions as the dependent variable. The regression model included the following study-level covariates selected a priori based on clinical relevance and availability across studies: mean age (continuous), study type (categorical: observational, retrospective cohort, and randomized controlled trial), energy type (categorical: including radiofrequency ablation and others), ablation site (categorical: left atrium, right atrium, and others), and follow-up duration in months (continuous). The regression was based on the random-effects meta-analysis model described previously and incorporated both within-study variance and between-study heterogeneity. The analysis was performed using the metareg function from the meta package in R. Between-study variance (τ^2^) in the meta-regression was estimated using the restricted maximum likelihood (REML) method, which provides unbiased estimates and performs well in the presence of moderate heterogeneity. Residual heterogeneity after the inclusion of covariates was assessed using τ^2^, I^2^, and H^2^ statistics. The proportion of between-study variance explained by the model was quantified using the pseudo-R^2^ metric. The overall significance of the moderators was tested using the omnibus Q-test for moderators (QM), and individual covariates were evaluated using Wald-type z-tests with 95% confidence intervals. A significance threshold of 0.05 was used for primary inference, with values between 0.05 and 0.10 interpreted as suggestive of potential associations.

A leave-one-out sensitivity analysis was conducted to evaluate the robustness of the pooled estimate to individual studies. Each study was systematically excluded, and the meta-analysis was re-estimated to identify potentially influential studies. Substantial shifts in the pooled effect size or τ^2^ were interpreted as evidence of study-level influence. In addition, subgroup analyses were performed to explore differences in recurrence rates across predefined study-level characteristics, including the energy type and ablation site. These subgroup estimates were derived using random-effects models within each stratum, and between-group heterogeneity was tested to assess effect modification.

Publication bias was assessed using multiple complementary approaches. A funnel plot was generated to visually inspect asymmetry in the distribution of effect sizes against their standard errors. In the absence of publication bias, studies are expected to scatter symmetrically around the pooled effect estimate, forming an inverted funnel shape. Deviations from this pattern may suggest small-study effects, including the selective publication of studies with significant or favourable results. To formally test for funnel plot asymmetry, Egger’s regression test was applied. This method regresses the standardized effect sizes on their standard errors and tests whether the intercept significantly deviates from zero, which would indicate asymmetry potentially due to publication bias. Additionally, Begg’s rank correlation test was used to evaluate the correlation between effect sizes and their variances; a significant result suggests systematic bias in the reporting of results. Rank correlation analysis (using Kendall’s τ) was also employed as a non-parametric measure of funnel plot asymmetry. This approach assesses whether there is a monotonic association between the standardized effect sizes and their precision ranks. Together, these methods provide a triangulated assessment of the likelihood of publication bias in the included literature.

R version 4.5.0 (R Foundation for Statistical Computing, Vienna, Austria) was utilized for all statistical analyses. OpenMeta[Analyst] (version 12.11.14) was used as a graphical user interface for the aforementioned R version and packages when generating the forest plots and leave-one-out analysis. The remaining work was completed using R Studio.

## 3. Results

### 3.1. Study Selection and Baseline Characteristics

The initial search yielded 1147 results. After removing duplicate records and ineligible studies, 67 remained and underwent full review based on the inclusion criteria. Of these, 37 studies were included, comprising 1453 patients from 34 observational cohort studies and 132 patients from 3 RCTs (Figure 1). The patients had a mean age of 40.8 ± 0.77 years, of whom 50.34% were female. The mean syncope recurrence rate was 2.29 ± 6.1, with a mean follow-up of 18 months. Other baseline patient and study characteristics are detailed in Table 1.

### 3.2. Pooled Analysis of the Primary Endpoint

**Figure 2:**
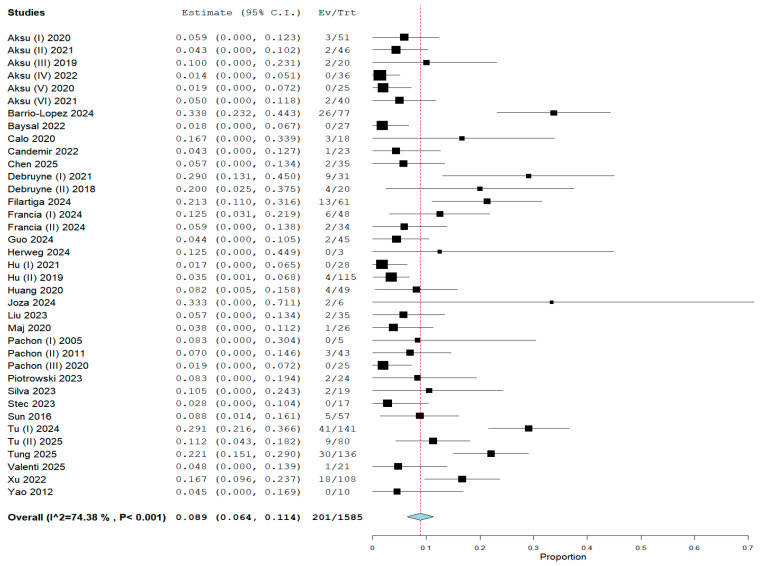
Forest plot of VVS recurrence with pooled rate of 8.9% after CNA [15,18,19,20,21,22,23,24,25,26,27,28,29,30,31,32,33,34,35,36,37,38,39,40,41,42,43,44,45,46,47,48,49,50,51,52,53].

In the pooled analysis, vasovagal syncope recurrence was reported in all 37 studies, encompassing 201 (12.53%) events from 1585 patients who underwent CNA. The overall weighted prevalence of syncope recurrence following CNA was 8.9% (95% CI: 6.4–11.4%; I^2^ = 74.38%) (Figure 2). We conducted a leave-one-out analysis to investigate the moderate-to-high heterogeneity of our primary outcome. This analysis reveals each study’s influence on heterogeneity and the pooled analysis (Figure S1).

#### 3.2.1. Subgroup Analysis of RCTs and Observational Studies on VVS Recurrence

**Figure 3:**
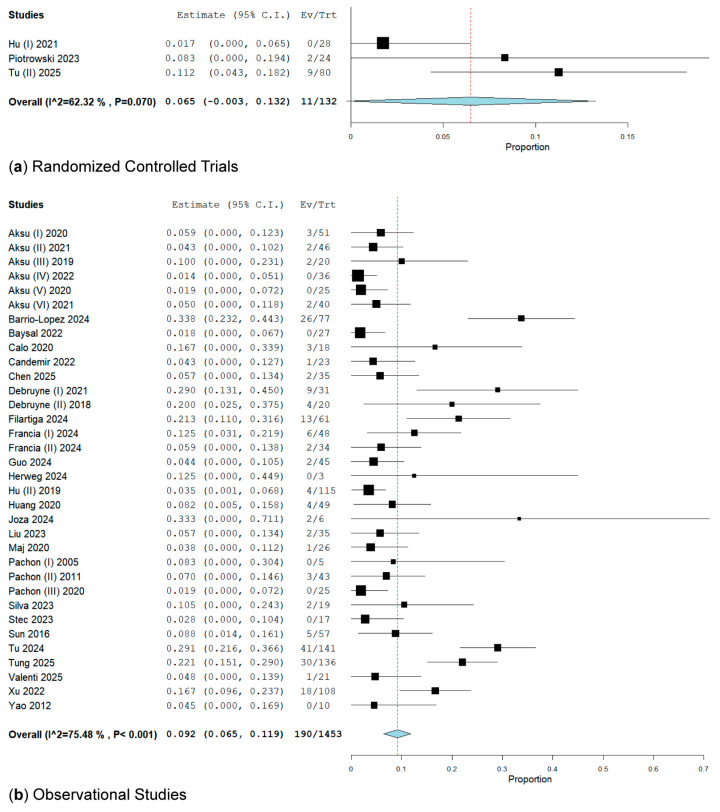
Forest plot of VVS recurrence after cardioneuroablation in (**a**) RCT [36,44,49] the pooled rate is 6.5%; (**b**) in observational studies [15,18,19,20,21,22,23,24,25,26,27,28,29,30,31,32,33,34,35,37,38,39,40,41,42,43,45,46,47,48,50,51,52,53] the pooled rate of recurrence is 9.2%.

In our subanalysis of RCTs, VVS recurrence was evidenced in three RCTs [36,44,49]. These RCTs involved 132 patients and reported 11 events (8.33%) with a rate of 6.5% (95% CI: 0.3–13.2%; I^2^ = 62.32%) (Figure 3).

In the observational studies group, which included 18 retrospective and 16 prospective cohort studies, VVS recurrence occurred in 190 patients out of a total of 1453, resulting in a rate of 9.2% (95% CI: 6.5–11.9%; I^2^ = 75.48%) (Figure 3).

#### 3.2.2. Subgroup Analysis of Energy Ablation Concerning the Primary Endpoint

**Figure 4:**
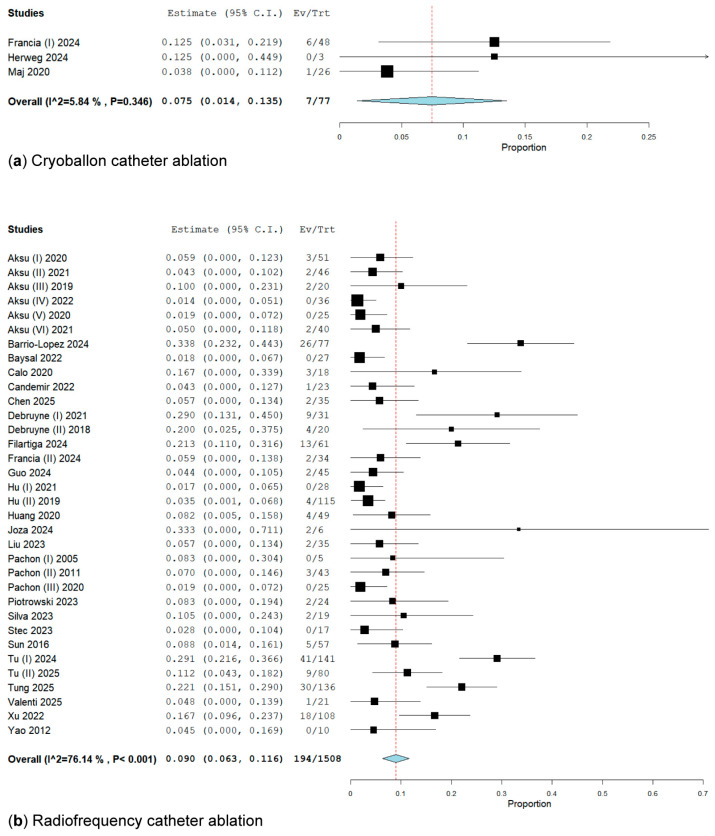
Forest plot of VVS recurrence on energy ablation subgroups. (**a**) Cryoballoon catheter ablation has a recurrence rate of 7.5% [32,35,41]; (**b**) radiofrequency catheter ablation has a recurrence rate of 9.09% [15,18,19,20,21,22,23,24,25,26,27,28,29,30,31,33,34,36,37,38,39,40,42,43,44,45,46,47,48,49,50,51,52,53].

In a subanalysis of types of energy by thermic ablation, in three studies [32,35,41] involving 77 patients who underwent cryoballoon ablation (CBA), 7 patients (9.09%) presented lower VVS recurrence at a rate of 7.5% (95% CI: 1.4–11.2%; I^2^ = 5.84%) compared to the radiofrequency ablation group [15,18,19,20,21,22,23,24,25,26,27,28,29,30,31,33,34,36,37,38,39,40,42,43,44,45,46,47,48,49,50,51,52,53], which included 194 of the 1508 patients, with a recurrence rate of 9.00% (95% CI: 6.3–11.6%; I^2^ = 76.14%) (Figure 4).

Our sensitivity analysis in trials that employed RFA for CNA, after excluding Tu (I) [48], Barrio-Lopez [24], Tung [50], Xu [52], Filartiga [31], and Debruyne (I) [29], revealed a rate of syncope recurrence of 8% (95% CI: 5.7–10.4%; I^2^ = 68.84%, *p* < 0.001), 7.1% (95% CI: 5–9.2%; I^2^ = 59.05%, *p* < 0.001), 6.1% (95% CI: 4.3–8%; I^2^ = 43.99%, *p* = 0.005), 5.5% (95% CI: 3.8–7.1%; I^2^ = 32.19%, *p* = 0.048), 4.8% (95% CI: 3.3–6.2%; I^2^ = 14.05%, *p* = 0.252), and 4.3% (95% CI: 3–5.6%; I^2^ = 0%, *p* = 0.662) (Figures S2–S12).

#### 3.2.3. Subgroup Analysis of CNA Location for the Primary Endpoint

**Figure 5:**
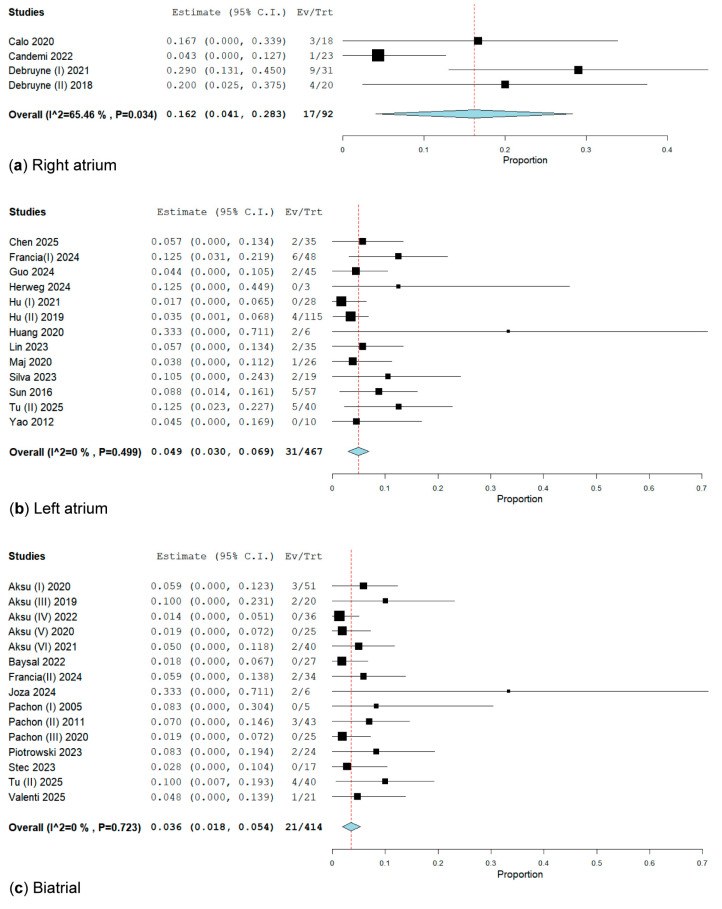
Forest plot of VVS recurrence by CNA localizations. (**a**) RA approach has 16.2% [26,27,29,30]; (**b**) LA approach has 4.9% [28,32,34,35,36,37,38,40,41,45,46,49,53]; (**c**) biatrial has the lowest recurrence rate of 3.6% [15,18,20,21,22,23,25,32,39,42,43,44,47,49,51].

We divided the studies based on the anatomical approach of CNA into three subgroups, as follows: only biatrial (475 patients from 16 studies), only LA (716 patients from 15 studies), and only RA (92 patients from 4 studies). We evaluated the impact of CNA location on VVS recurrence, as follows: 7.15% from biatrial, 12.56% from LA, and 18.47% from RA (Figure 5).

In the subgroup analysis of RA location for CNA, we included four studies [26,27,29,30] involving a total of 92 patients, with 17 experiencing VVS recurrence, resulting in a rate of 16.2% (95% CI: 4.1–28.3%; I^2^ = 65.46%, *p* = 0.034) (Figure 5). We performed a sensitivity analysis on trials utilizing the RA approach for CNA. After excluding Candemir [27], the syncope rate was 22.3% (95% CI: 12.6–32%; I^2^ = 0%, *p* = 0.560) (Figures S13 and S14).

In the subgroup analysis of LA location for CNA, which included 15 studies [28,32,34,35,36,37,38,40,41,45,46,49,53] involving a total of 716 patients, 90 of whom had VVS recurrence, the rate was 9.3% (95% CI: 5.1–13.4%; I^2^ = 75.1%, *p* = 0.001) (Figure S15). We performed a sensitivity analysis in trials utilizing the LA approach for CNA. After excluding Tu (I) [48] and Xu [52], the syncope rate was 6.8% (95% CI: 4.1–9.5%; I^2^ = 38.9%, *p* = 0.068) (Figure S17) and, respectively, 4.9% (95% CI: 3–6.9%; I^2^ = 0%, *p* = 0.499) (Figure 5).

The subgroup analysis of the biatrial location of CNA included 16 studies [15,18,20,21,22,23,25,32,39,42,43,44,47,49,51] with a total of 475 patients, from which 34 patients had VVS recurrence with a rate of 4.9% (95% CI: 2.6–7.1%; I^2^ = 30.58%, *p* = 0.119) (Figure S19). After a sensitivity analysis excluding Filartiga [31], the syncope rate was 3.6% (95% CI: 1.8–5.4%; I^2^ = 0%, *p* = 0.723) (Figure 5).

#### 3.2.4. Subgroup Analysis by Age

**Figure 6:**
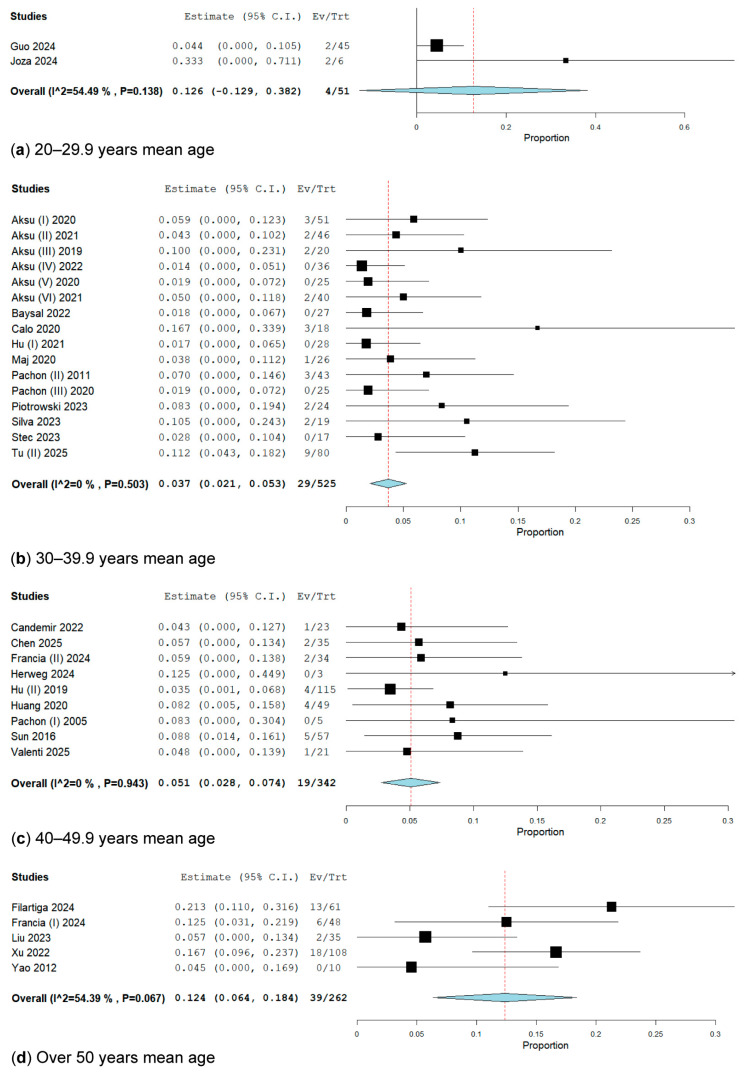
Forest plot of VVS recurrence on age subgroups. (**a**) 20–29.9 years, mean age has 12.6% [34,39]; (**b**) 30–39.9 years, mean age has 3.7% [15,18,19,20,21,22,23,25,26,36,41,43,44,46,47,49]; (**c**) 40–49.9 years, mean age has 5.1% [27,28,32,35,37,42,45,51]; (**d**) over 50 years, mean age has 12.4% [31,32,40,52,53].

We divided the population into four categories based on mean age, as follows: patients with a mean age of 20 to 29.9 years, comprising 2 studies; subgroup 2, with a mean age of 30 to 39.9 years, comprising 17 studies; with a mean age of 40 to 49.9 years, comprising 12 studies; and patients with a mean age of over 50 years, comprising 5 studies (Figure 6).

In the subgroup analysis of patients with a mean age of 20 to 29.9 years, which included two studies [34,39] with 51 patients, only 4 patients exhibited VVS recurrence. The overall weighted recurrence rate of VVS was 12.6% (95% CI: 12.9–38.2%; I^2^ = 54.49%, *p* = 0.138) (Figure 6).

In the subgroup analysis of patients aged between 30 and 39.9 years, which included 17 studies [15,18,19,20,21,22,23,25,26,36,41,43,44,46,47,49] involving 556 patients, 38 patients experienced recurrent VVS. The overall weighted recurrence rate of VVS was 4.6% (95% CI: 2.5–6.6%; I^2^ = 32.99%, *p* = 0.092). After conducting a sensitivity analysis, we excluded Debruyne (I) [29], resulting in a recurrence rate of 3.7% (95% CI: 2.1–5.3%; I^2^ = 0%, *p* = 0.503) (Figure 6).

In the subgroup analysis of patients with a mean age between 40 and 49.9 years, which included 12 studies involving 696 patients, 116 presented with VVS recurrence. The overall weighted VVS recurrence rate was 12.2% (95% CI: 6.1–18.3%; I^2^ = 85.49%, *p* < 0.001) (Figure S22). We conducted a sensitivity analysis in these trials. After excluding Barrio-Lopez [24], Tu (I) [48], and Tung [50], the VVS recurrence rates were 10.2% (95% CI: 4.6–15.8%; I^2^ = 81.73%, *p* < 0.001) (Figure S24), 7.9% (95% CI: 3.8–12.1%; I^2^ = 61.51%, *p* = 0.005) (Figure S26), and 5.1% (95% CI: 2.8–7.4%; I^2^ = 0%, *p* = 0.943) (Figure 6).

In the subgroup analysis of patients with a mean age over 50 years, which included five studies [31,32,40,52,53] with a total of 262 patients, of whom 39 experienced VVS recurrence, the overall weight rate of VVS recurrence was 12.4% (95% CI: 6.4–18.4%; I^2^ = 54.39%, *p* = 0.067) (Figure 6).

#### 3.2.5. Subanalysis on an Additional Technique for Electrical Anatomical Mapping (EAM) When Performing GP Ablation

**Figure 7:**
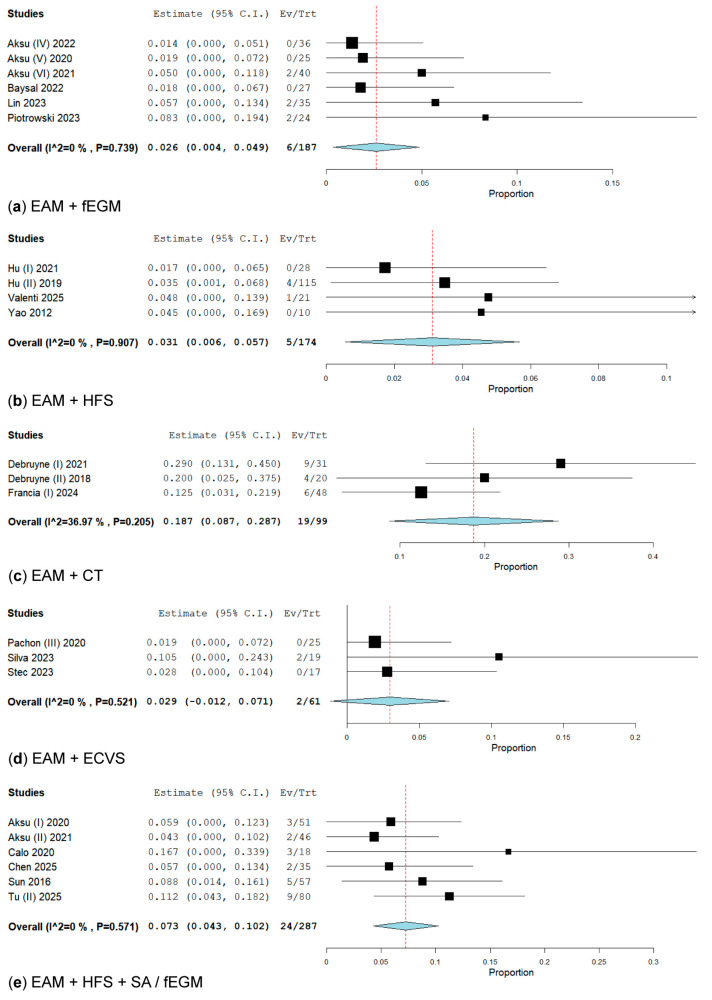
Forest plot on VVS recurrence using additional technique for EAM. (**a**) EAM + fEGM [21,22,23,25,40,44]; (**b**) EAM + HFS [36,37,51,53]; (**c**) EAM + CT [29,30,32]; (**d**) EAM + ECVS [43,46,47]; (**e**) EAM + HFS + SA/fEGM [18,19,26,28,45,49].

Six studies [21,22,23,25,40,44] included EAM + fEGM, involving 187 patients, of whom 6 presented with VVS recurrence, resulting in a rate of 2.6% (95% CI: 0.4–4.9%; I^2^ = 0%, *p* = 0.739) (Figure 7). Four studies [36,37,51,53] used EAM + HFS, with 174 patients, of whom only 5 had VVS recurrence, yielding a rate of 3.1% (95% CI: 0.6–5.7%; I^2^ = 0%, *p* = 0.907) (Figure 7). Three studies [29,30,32] utilised EAM + CT, including a total of 99 patients, of whom 9 experienced VVS recurrence, indicating a rate of 18.7% (95% CI: 8.7–28.7%; I^2^ = 36.97%, *p* = 0.205) (Figure 7). Three studies [43,46,47] incorporated ECVS into EAM, totalling 61 patients, among whom 2 had VVS recurrence, reflecting a rate of 2.9% (95% CI: 1.2–7.1%; I^2^ = 0%, *p* = 0.521) (Figure 7). Lastly, six studies [18,19,26,28,45,49] conducted EAM with HFS + SA/fEGM, encompassing 287 patients, with 24 experiencing VVS recurrence, resulting in a rate of 7.3% (95% CI: 4.3–10.2%; I^2^ = 0%, *p* = 0.571) (Figure 7).

### 3.3. Secondary Outcomes Analysis

#### 3.3.1. Heart Rate After Cardioneuroablation

**Figure 8:**
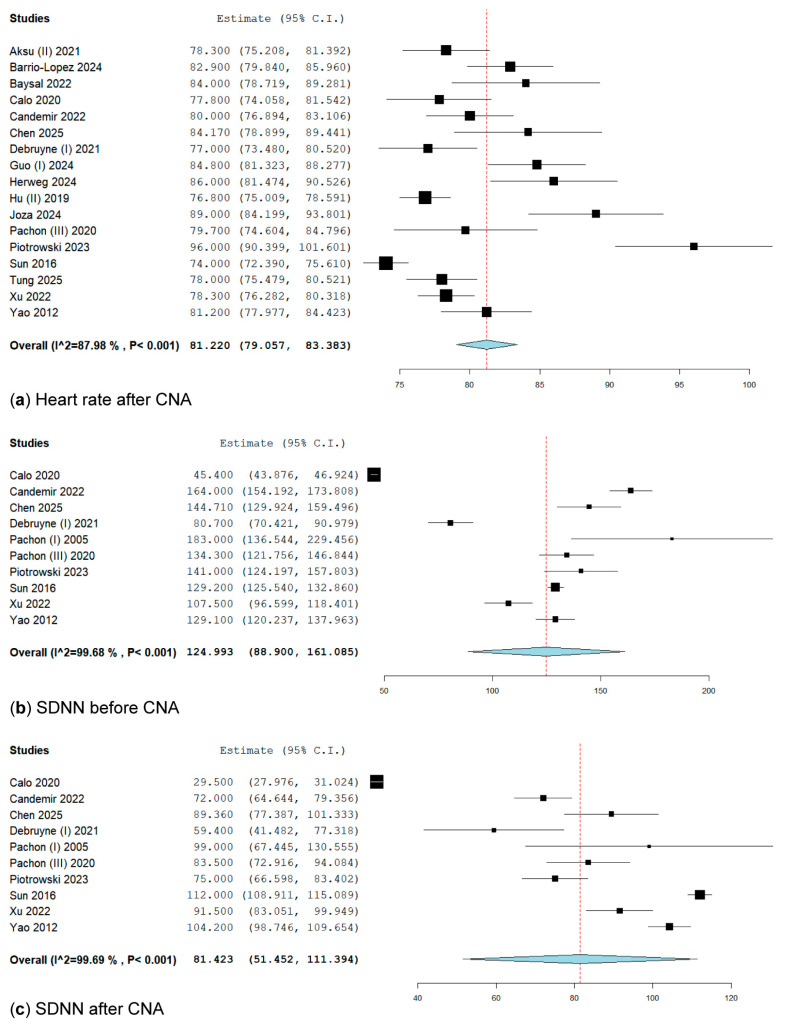
Forest plots of secondary outcomes. (**a**) Heart rate after CNA is 81.22 msec [19,24,25,26,27,28,29,34,35,37,39,43,44,45,50,52,53]; (**b**) SDNN before CNA is 124.99 msec [26,27,28,29,42,43,44,45,52,53]; (**c**) SDNN after CNA is 81.42 msec [26,27,28,29,42,43,44,45,52,53].

In the pooled analysis, heart rate following cardioneuroablation was reported in 17 studies [19,24,25,26,27,28,29,34,35,37,39,43,44,45,50,52,53], encompassing 786 patients who underwent CNA. The overall weighted mean HR after CNA was 81.22 bpm (95% CI: 79.05–83.38; I^2^ = 87.98%) (Figure 8). Given the high heterogeneity in our secondary outcome, we conducted a leave-one-out analysis to explore our findings. The results of this analysis illustrate each study’s influence on heterogeneity and pooled analysis (Figure S29).

#### 3.3.2. SDNN Before Cardioneuroablation

The SDNN before CNA was reported in ten studies [26,27,28,29,42,43,44,45,52,53] involving 336 patients. The overall weighted mean was 124.99 msec (95% CI: 88.9–161.08; I^2^ = 99.68, *p* < 0.001) (Figure 8). We conducted a leave-one-out analysis to investigate our findings, which revealed very high heterogeneity in our secondary outcome. The results of this analysis demonstrate each study’s influence on heterogeneity and the pooled analysis (Figure S30).

#### 3.3.3. SDNN After Cardioneuroablation

Regarding the SDNN after CNA, reported in the same 10 studies, the overall weighted mean was 81.42 msec (95% CI: 51.45–111.39; I^2^ = 99.69, *p* < 0.001) (Figure 8). We conducted a leave-one-out analysis to investigate our findings, demonstrating high heterogeneity in our secondary outcome. This analysis reveals each study’s influence on heterogeneity and the pooled analysis (Figure S31).

#### 3.3.4. Reintervention Rate

**Figure 9:**
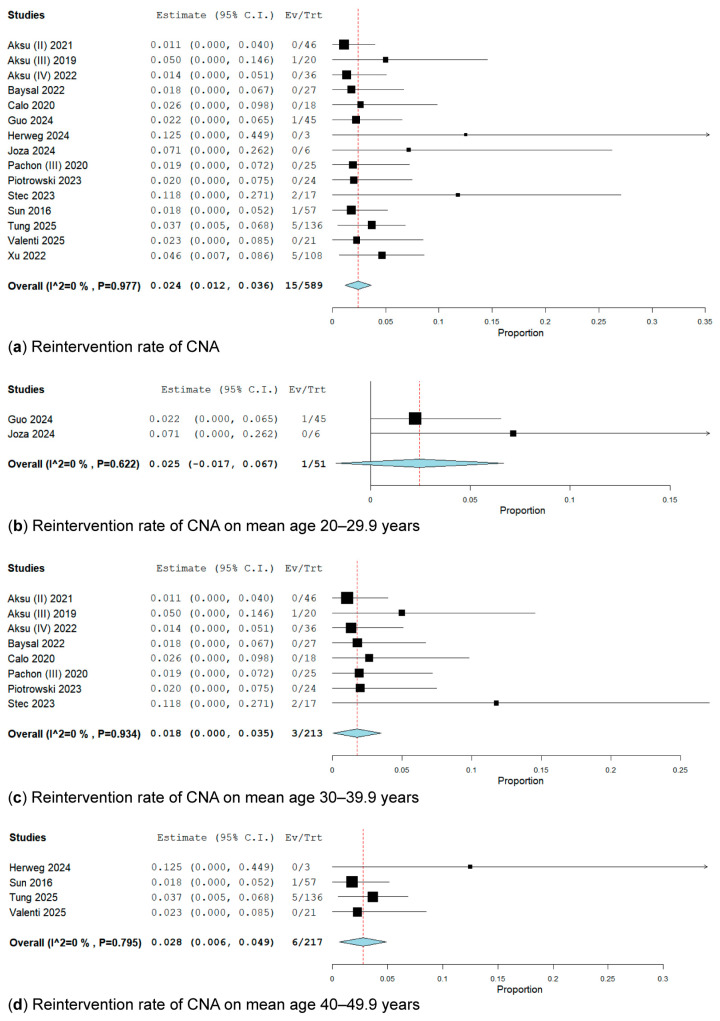
(**a**) Forest plots of reintervention rate of CNA [19,20,21,25,26,34,35,39,43,44,45,47,50,51,52]. Forest plots of CNA reintervention subgroups of mean age: (**b**) 20–29.9 years [34,39]; (**c**) 30–39.9 years [19,20,21,25,26,43,44,46]; (**d**) 40–49.9 years [35,47,50,51].

In the pooled analysis, the reintervention rate of cardioneuroablation was reported in 15 studies [19,20,21,25,26,34,35,39,43,44,45,47,50,51,52] encompassing 589 patients, of whom only 15 underwent a redo-CNA. The overall weighted prevalence of redo-CNA was 2.4% (95% CI: 12–36%; I^2^ = 0%, *p* = 0.977) (Figure 9).

We perform a subgroup analysis of the reintervention rate of cardioneuroablation based on mean age (Figure 9); therefore, we divided the studies into the following three subgroups: 20–29.9 years [34,39], 30–39.9 years [19,20,21,25,26,43,44,46], and 40–49.9 years [35,47,50,51] (Figure 9).

In the subgroup analysis of patients with a mean age between 20 and 29.9 years, which included two studies [34,39] comprising 51 patients, only one patient underwent a redo-CNA for VVS recurrence. The overall rate of reintervention was 2.5% (95% CI: 1.7–6.7%; I^2^ = 0%, *p* = 0.622).

In the subgroup analysis of patients with a mean age between 30 and 39.9 years [19,20,21,25,26,43,44,46], which included eight studies encompassing 213 patients, only 3 patients underwent a redo-CNA for VVS recurrence. The overall weighted reintervention rate was 1.8% (95% CI: 0.0–3.5%; I^2^ = 0%, *p* = 0.934).

In the subgroup analysis of patients with a mean age between 40 and 49.9 years, which included four studies [35,47,50,51] involving 217 patients, only 6 presented with a redo-CNA for VVS recurrence. The overall weighted reintervention rate was 2.8% (95% CI: 0.6–4.9%; I^2^ = 0%, *p* = 0.795).

### 3.4. Additional Heterogeneity Analysis: Meta-Regression and Baujat Plot

A mixed-effects meta-regression was conducted to explore whether between-study heterogeneity in recurrence rates could be explained by key study-level covariates. The model included the following predictors: mean age (continuous), study type (observational, RC, or RCT), energy type (categorical), ablation site (LA, RA, or BA), and follow-up duration in months (continuous). The regression was applied to arcsine-transformed proportions using the REML method for estimating residual heterogeneity (τ^2^).

The meta-regression model included 37 studies and yielded a τ^2^ value of 0.0233 (SE = 0.0084), indicating moderate residual heterogeneity after accounting for covariates. The corresponding I^2^ was 78.18%, meaning that approximately 78% of the total observed variability was due to true heterogeneity not attributable to the sampling error. The H^2^ value of 4.58 suggests that the total variability was 4.58 times greater than would be expected from within-study error alone. The R^2^ value was 4.69%, indicating that the included moderators jointly explained only a small portion of the between-study heterogeneity.

The test for residual heterogeneity (QE) remained highly significant (QE = 125.38, df = 29, *p* < 0.0001), confirming that substantial heterogeneity persisted even after adjusting for the included covariates. The test for the joint effect of moderators (QM) was not statistically significant (QM = 6.48, df = 7, *p* = 0.4853), suggesting that the covariates as a group did not explain a substantial proportion of the variability in recurrence rates.

At the individual covariate level, several variables showed borderline statistical signals. Mean age had a positive association with recurrence (estimate = 0.0080, SE = 0.0048, z = 1.67, *p* = 0.0955), suggesting a trend toward higher recurrence with increasing age, though this did not reach conventional significance. Similarly, ablation at the RA showed a positive association with recurrence (estimate = 0.1774, SE = 0.1046, z = 1.70, *p* = 0.0899), again indicating a possible effect that was not statistically definitive. All other variables, including study type, energy type, left atrial ablation, and follow-up duration, showed non-significant associations with recurrence, with wide confidence intervals that crossed zero.

In summary, the meta-regression did not identify any statistically significant predictors of recurrence at the conventional alpha level of 0.05. Although mean age and right atrial ablation approached significance, the model explained only a small proportion of the heterogeneity, and substantial residual variability remained unexplained. These results suggest that additional, unmeasured factors may be contributing to heterogeneity in recurrence outcomes across studies.

A Baujat plot (Figure S34) was generated to identify individual studies that have a disproportionate influence on the pooled effect size and/or contribute to between-study heterogeneity. Most studies clustered in the lower-left quadrant of the plot, indicating minimal influence on the overall result and low contribution to heterogeneity, suggesting general consistency across the dataset.

However, two studies emerged as influential outliers. Tu (I) [48] contributed most substantially to the overall heterogeneity, with a Q-contribution of approximately 48, indicating it was the single largest driver of observed heterogeneity. Barrio-Lopez [24] had the greatest influence on the pooled effect size (influence ≈ 0.12) and also contributed meaningfully to between-study heterogeneity. Additional studies, including Aksu (IV) [21], Tung [50], and Filartiga [31], demonstrated a moderate impact in one or both dimensions, remaining within an acceptable range.

### 3.5. Quality Assessment

**Figure 10:**
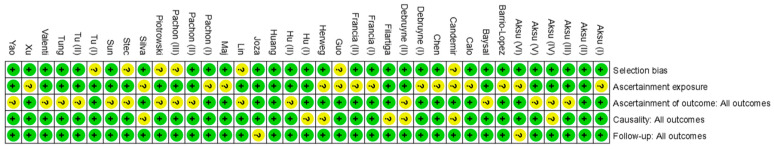
Risk of bias for quality assessment of included studies [15,18,19,20,21,22,23,24,25,26,27,28,29,30,31,32,33,34,35,36,37,38,39,40,41,42,43,44,45,46,47,48,49,50,51,52,53].

The Newcastle–Ottawa tool was employed for quality assessment. No studies were deemed at a high risk of bias, as illustrated in Figure 10. Each study was examined concerning the following criteria: selection (Do the patients represent the entire experience of the investigator (centre), or is the selection method ambiguous to the point that other patients with similar presentations may not have been included?), ascertainment (Was the exposure adequately determined? Was the outcome adequately determined?), and causality (Were alternative causes that could explain the observation ruled out? Was the follow-up period sufficient for outcomes to manifest?). In the risk of bias summary, studies exhibited symmetrical distribution based on weight and converged on the pooled effect as the weight increased.

## 4. Discussion

Our updated systematic review and meta-analysis, involving 37 studies and 1585 patients with VVS, examined the effectiveness and safety of CNA and its impact on autonomic function. The pooled recurrence rate following CNA was 8.9% (95% CI: 6.4–11.4%), indicating high procedural efficacy. Our findings align broadly with earlier studies by Vandenberk et al. (2022) [54,55], who reported a syncope recurrence rate of 8.1%, and Prata et al. (2024) [55], who noted a slightly lower recurrence rate of 5.94%. Compared to these studies, our updated review features a larger patient sample, a wider array of study designs, and more recent publications extending through 2025, enhancing our findings’ external validity and relevance. Our recurrence estimates remained robust across sensitivity analyses, including leave-one-out tests.

One significant improvement in this meta-analysis is including and evaluating physiological outcomes linked to autonomic modulation. We found a consistent rise in resting heart rate after the procedure and a decrease in SDNN, both signs of reduced parasympathetic tone. These findings back up the idea that CNA achieves clinical benefits through selective vagal denervation, particularly at the level of atrial GPs. In contrast, the Vandenberk et al. (2022) [54] and Prata et al. (2024) [55] meta-analyses both highlighted the importance of GP modulation; however, neither provided pooled estimates of post-ablation SDNN or heart rate. Our study fills this gap, offering quantifiable insights into the mechanistic effects of CNA beyond just symptom recurrence.

Subgroup analyses revealed significant differences in efficacy based on the anatomical site of ablation. Procedures targeting only the right atrium were associated with a recurrence rate of 15.78%, substantially higher than those targeting the left atrium (5.3%) or biatrial (4.17%). These findings align with the Prata et al. (2024) [55] study, which also reported fewer effective outcomes for RA-only ablation. Additional evidence supports the growing consensus that biatrial and left atrial ablation methods are associated with lower recurrence rates compared to right atrial ablation. However, due to the observational nature of the included studies, these findings should be interpreted with caution. In contrast, the earlier meta-analysis by Vandenberk et al. (2022) [54] did not identify significant differences by ablation site, possibly due to smaller sample sizes and limited subgroup data. By expanding the available dataset and refining subgroup definitions, our analysis provides a clearer understanding of this important procedural variable.

Energy modality has emerged as another factor influencing treatment response. Although RFA remains the most commonly used technique, our subgroup analysis revealed a recurrence rate of just 7.5% (95% CI: 1.4–13.5%) in patients treated with CBA. While this finding should be interpreted with caution due to the limited number of CBA studies (n = 3), it suggests comparable or possibly improved outcomes compared to RFA. This observation differs slightly from Prata et al. (2024) [55], who did not identify CBA as a distinct subgroup, and it was not addressed by Vandenberk et al. (2022) [54], whose analysis was conducted before CBA-based CNA became more widely used. The potential for CBA to offer consistent lesion sets with favourable safety profiles warrants further investigation.

Post-procedural increases in heart rate were consistently reported, with an average rise from around 63 bpm to over 81 bpm. Similarly, SDNN values decreased significantly from about 125 ms to approximately 81 ms, underscoring the physiological impact of CNA on autonomic balance. These autonomic changes were not highlighted in previous meta-analyses, making our findings distinctive in their focus on the potential role of heart rate variability as a surrogate marker for effective denervation. Since CNA relies on modulating neural input rather than mechanical pacing, these results help define the physiological end goals of the procedure and may serve as intra- or post-procedural success metrics in future studies.

Finally, our analysis of recurrence and reintervention rates by age group found no significant differences between younger and older patients, suggesting that CNA may be effective across a wide age range. This challenges earlier assumptions that the procedure should only be used in younger patients with pure cardioinhibitory reflexes and widens its potential use. Neither Vandenberk et al. (2022) [54] nor Prata et al. (2024) [55] examined age-stratified outcomes in detail, making our review a significant contribution to the evolving clinical landscape of CNA. These findings provide a robust evidence base to support the individualized, anatomy-specific, and physiology-guided use of CNA in treating recurrent VVS.

## 5. Limitations, Strengths, and Future Directions

Our systematic review and meta-analysis have several key strengths that enhance their reliability and relevance to clinical practice. Firstly, our comprehensive investigation encompasses 37 studies and 1585 patients—the largest pooled sample in the field to date—and integrates studies published up to 2025. This expanded dataset enables a more precise and generalizable estimate of CNA outcomes in patients with VVS. Secondly, we conducted a multidimensional analysis that extends beyond simple recurrence rates. Unlike previous reviews, we also examined autonomic parameters, such as heart rate and the SDNN, as well as post-procedural reintervention rates and procedural safety outcomes. This broader set of clinical endpoints affords a more holistic understanding of CNA’s therapeutic impact. Thirdly, detailed subgroup and sensitivity analyses bolster the robustness of our findings. We stratified the data based on ablation energy modality, the anatomical location of ganglionated plexi ablation, and patient age, while also employing leave-one-out sensitivity testing to evaluate the influence of individual studies on pooled estimates. These rigorous analytic strategies enhance the credibility of our conclusions. Ultimately, methodological quality was thoroughly considered. All included studies underwent appraisal using the Newcastle–Ottawa Scale, and none were deemed to be at high risk of bias. Furthermore, funnel plot analyses (Figure S32) and statistical tests (Figure S33) indicated significant asymmetry, suggesting the presence of publication bias or small-study effects. This may have led to an overestimation of the pooled effect size, reinforcing the need for cautious interpretation of the results, especially in the context of observational data.

Several limitations must be acknowledged. The evidence base consists primarily of observational studies (34 out of 37), which limits the ability to draw strong causal inferences. Although observational data can provide useful insights, they are inherently prone to confounding, selection bias, and reporting variability. Only three randomized controlled trials were included, underscoring the need for further high-quality interventional studies to validate the observed effects of CNA. The studies showed significant variations in methodology, including differences in patient populations, ablation techniques, procedural endpoints, and outcome definitions. The interpretation of changes in HR and HR variability parameters, such as SDNN, is further limited by the lack of standardization in measurement protocols across studies. Variability in recording duration (e.g., 5 min vs. 24 h), patient activity level, and analysis software may contribute to heterogeneity and limit the comparability of results.

The evaluation of autonomic function was not standardized across studies. Significant methodological variability was observed in the type of autonomic tests performed (e.g., tilt-table testing, HR variability via ECG or Holter, deep breathing tests), the duration and timing of measurements post-procedure, and the conditions of data acquisition (e.g., rest vs. activity). These inconsistencies limit the comparability of results and introduce potential measurement bias in pooled secondary outcomes. Standardized autonomic testing protocols are needed in future trials to improve data quality and interpretability.

These several secondary outcomes, such as heart rate variability parameters (e.g., SDNN), were derived from a small number of studies with limited sample sizes and high between-study variance. Similarly, some subgroup analyses (e.g., RCT, mean age 20–29.9, RA-only ablation) included only two or three studies, making them vulnerable to small-study effects and limiting their reliability. These findings should be interpreted with caution and considered exploratory. Furthermore, although the mean follow-up duration was 18 months, data on outcomes beyond 36 months were scarce. This restricts our ability to evaluate the long-term durability of CNA and the potential for late recurrences. Additionally, the non-standardized nature of autonomic measurements limits our ability to draw firm conclusions about the physiological impact of CNA. Variation in how SDNN and heart rate were assessed before and after ablation introduces interpretive challenges. Lastly, the single-arm design of this meta-analysis restricts comparative effectiveness insights. Without a control group, such as patients receiving standard medical therapy or pacemaker implantation, we cannot definitively assess how CNA performs relative to alternative treatments.

Future research should build on the findings from this meta-analysis to address the current gaps in evidence. First, there is a pressing need for multicentre, double-blind, randomized controlled trials to directly compare CNA with placebo or standard therapies, such as pacemaker implantation. These trials would provide high-level evidence to inform guideline development and clinical decision making. Second, there is a need for consensus on procedural endpoints, including standardized definitions for procedural success, consistent GP targeting methods, and objective intra-procedural markers like vagal response testing. A unified approach would greatly enhance reproducibility and comparability across studies. Third, researchers should investigate long-term autonomic remodelling after CNA using validated physiological biomarkers and novel tools like wearable monitors. Understanding how CNA alters autonomic tone over time may offer predictive insights into recurrence and response durability. Fourth, patient-level meta-analyses could help identify predictors of recurrence, especially in complex subgroups like older patients or those with mixed or vasodepressor VVS types. Tailoring the intervention based on these predictors may improve individual patient outcomes. Lastly, several practical barriers limit the widespread adoption of CNA in routine care. The cost-effectiveness of CNA remains uncertain, particularly given the need for specialized equipment, extended procedural time, and follow-up. There is a lack of procedural standardization, with considerable variation in anatomical targets, ablation endpoints, and mapping techniques across studies. Finally, inter-operator variability may significantly influence procedural outcomes, as CNA is technically demanding and not yet widely practised. These issues underscore the importance of future prospective trials designed not only to evaluate clinical outcomes but also to address implementation feasibility, training, and cost-utility.

## 6. Conclusions

Our updated systematic review and single-arm meta-analysis shows that cardioneuroablation is linked to a low recurrence rate for vasovagal syncope and positive changes in autonomic regulation, especially when targeting the left or biatrial ganglionated plexi. These results suggest that cardioneuroablation may be a viable treatment option for selected patients with recurring or treatment-resistant vasovagal syncope. Although our findings suggest CNA is a promising therapeutic approach, especially in procedures guided by anatomy and physiology, more randomized controlled trials are needed to confirm its long-term effectiveness, refine procedural outcomes, and clarify patient selection criteria.

## Figures and Tables

**Table 1:** Baseline characteristics of included studies [15,18,19,20,21,22,23,24,25,26,27,28,29,30,31,32,33,34,35,36,37,38,39,40,41,42,43,44,45,46,47,48,49,50,51,52,53]. Abbreviations: randomised controlled trial (RCT), retrospective cohort (RC), prospective cohort (PC), vasovagal syncope (VVS), cardioneuroablation (CNA), radiofrequency ablation (RFA), cryoballoon ablation (CBA), pulmonary vein isolation (PVI), electroanatomic mapping (EAM), high-frequency stimulation (HFS), fractionated electrograms (fEGM), spectral analysis (SA), extracardiac vagal stimulation (ECVS), computerised tomography (CT), right atrium (RA), left atrium (LA), biatrial (BA), heart rate (HR), standard deviation of normal-to-normal intervals (SDNN), high-density fractionation mapping (HDFM), recurrent (R), cardioinhibitory (CI), mixed (M), vasodepressor (V).

Study and Year	Design	Sample Size	Location of Ablation	Type of Ablation	Method of Ablation	Age	Female, n (%)	Type of Syncope	Syncope’s Burden	Follow-up (Months)
Aksu (I) (2020) [18]	RCT	51	BA	RFA	EAM/HFS + SA	35.5 ± 12.2	19 (37.25)	R	5.5 ± 2.9	22.2
Aksu (II) (2021) [19]	PC	46	BA	RFA	EAM + HFS + SA fEGM	39.4 ± 14	27 (41.5)	R	4.7 ± 3	14.9
			RA	RFA	HFS + SA/EAM					
Aksu (III) (2019) [20]	PC	20	BA	RFA	EAM + fEGM	36 ± 12.8	10 (50)	R	NA	24
				RFA	EAM + HFS					
Aksu (IV). (2022) [21]	RC	36	BA	RFA	EAM + fEGM	35.1 ± 14	19 (52.77)	R	6.7 ± 4	8
Aksu (V) (2020) [22]	RC	25	BA	RFA	EAM + fEGM	39.6. ± 14	16 (32.7)	R	2.8 ± 1.9	9.5
Aksu (VI) (2021) [23]	RC	40	BA	RFA	EAM + fEGM	36 ± 13	17 (42.5)	R, CI	6.2 ± 3.4	12.1
Barrio-Lopez (2024) [24]	RC	77	BA, RA, LA	RFA	EAM + fEGM + HFS + ECVS	49.3 ± 13.4	42 (54.5)	R, CI, M	6.9 ± 3.11	6
Baysal (2022) [25]	PC	27	BA	RFA	EAM + fEGM	34 ± 14	13 (48.14)	R	5.5 ± 5.56	12
Calo (2020) [26]	PC	18	RA	RFA	EAM + fEGM + HFS + SA	36.9 ± 11.2	10 (55.55)	CI	5.2 ± 1.4	34.1
Candemir (2022) [27]	RC	23	RA	RFA	EAM	40.7 ± 13.2	10 (43.47)	R, CI, M	3.3 ± 1.3	10
Chen (2025) [28]	RC	35	LA	RFA	HFS + EAM	47.48 ± 16.49	17 (48.57)	R, CI, M, V	4.14 ± 4.45	11
Debruyne (I) (2021) [29]	PC	31	RA	RFA	EAM + CT	39.6 ± 9	16 (51.61)	R	13.3 ± 22.3	12
Debruyne (II) (2018) [30]	PC	20	RA	RFA	EAM + CT	NA	6 (50)	M, CI	NA	8.7
Filartiga (2024) [31]	PC	61	BA	RFA	EAM	50 ± 15	24 (39)	NA	NA	16
			BA							19
Francia (I) (2024) [32]	RC	48	LA	CBA	EAM + CT	51 ± 16	23 (47.91)	R	NA	10.2
Francia (II) (2024) [33]	RC	34	BA	RFA	EAM + CT + fEGM	47.9 ± 17.2	11 (32.35)	R	4.1 ± 4.03	24.5
Guo (2024) [34]	RC	45	LA	RFA	EAM	22.5 ± 4.4	2 (4.44)	R	3.44 ± 1	15
				RFA	HFS					
Herweg (2024) [35]	PC	3	LA	CBA	EAM	48 ± 14.7	1 (33.33)	R	2.6 ± 0.6	5.94
Hu (I) (2021) [36]	RCT	28	LA	RFA	EAM + HFS	39.5 ± 11.06	20 (71.42)	R	5 ± 3.51	16.5
Hu (II) (2019) [37]	RC	115	LA	RFA	EAM + HFS	42.9 ± 17.9	69 (60)	R	3.5 ± 4.4	21.4
Huang (2020) [38]	PC	49	LA	RFA	EAM	42.4 ± 16.1	27 (55.1)	R	NA	16.4
Joza (2024) [39]	PC	6	BA	RFA	EAM + fEGM + HFS	29 ± 4	4 (67)	CI	14 ± 7.2	13.4
Lin (2023) [40]	RC	35	LA	RFA	EAM + fEGM	53.9 ± 13.6	42 (60)	R, CI, M, V	9.6 ± 15.4	12
Maj (2020) [41]	RC	26	LA	CBA	NA	37.5 ± 9.0	6 (23.1)	CI	2.6 ± 0.8	20.1
Pachon (I) (2005) [42]	RC	5	BA	RFA	EAM + SA	47.5 ± 16	NA	CI	NA	9.2
Pachon (II) (2011) [15]	PC	43	BA	RFA	EAM + SA	32.9 ± 15	18 (41.9)	M, CI	4.7 ± 2	45.1
Pachon (III) (2020) [43]	PC	25	BA	RFA	EAM + ECVS	36.3 ± 19	40 (48)	R	3.8 ± 2	40
Piotrowski (2022) [44]	RCT	24	BA	RFA	EAM + fEGM	38 ± 10	26 (54.16)	R	3 ± 3	24
Sun (2016) [45]	PC	57	LA	RFA	EAM + HFS	43.2 ± 13.4	35 (61.4)	R	3 ± 0.87	36.4
Silva (2023) [46]	PC	19	LA	RFA	EAM + ECVS	37.8 ± 12.9	6 (32)	R	NA	21.0
Stec (2023) [47]	PC	17	BA	RFA	EAM + ECVS	37.7 ± 1.47	8 (40)	CI	NA	20.8
Tu (I) (2024) [48]	RC	141	LA	RFA	EAM + HFS	40 ± 18	90 (63.82)	R, CI, M, V	2.6 ± 2.22	51.6
Tu (II) (2025) [49]	RCT	80	BA	RFA	EAM + HFS + SA/fEGM	38 ± 16	43 (53.8)	M, CI	3.06 ± 0.62	12
			LA	RFA	HFS + SA/EAM					
Tung (2025) [50]	RC	136	BA, RA, LA	RFA	HFS + HDFM	47.4 ± 17	18 (41.9)	R, M, V	7.16 ± 2	14
Valenti (2025) [51]	RC	21	BA	RFA	EAM + HFS	42 ± 21	8 (38)	R	NA	12
Xu (2022) [52]	RC	108	LA	RFA	EAM	51.2 ± 15.3	48 (44.44)	M, V	2.58 ± 1.77	10
				RFA	HFS					
Yao (2012) [53]	PC	10	LA	RFA	EAM + HFS	50.4 ± 6.4	7 (70)	R	7.1 ± 13.33	30

## Data Availability

This study is based on previously published data. No new data were generated or analysed.

## Supplementary Materials

The following supporting information can be downloaded at: https://www.mdpi.com/article/10.3390/biomedicines13071758/s1, Figure S1–S31: Forest plots from various sensitivity analyses assessing the robustness of results regarding vasovagal syncope recurrence after cardioneuroablation, with subgroup analyses by ablation technique (CBA, RFA), anatomical localization (right atrium, left atrium, biatrial), age stratification, and heart rate variability metrics; Figure S32–S34: Funnel plot of assessment publication bias, asymmetry tests, and Baujat plot.

## Abbreviations

The following abbreviations are used in this manuscript:

AV	Atrioventricular
BA	Biatrial
BPM	Beats per minute
CBA	Cryoballoon ablation
CI	Cardioinhibitory
CNA	Cardioneuroablation
CT	Computerised tomography
ECVS	Extracardiac vagal stimulation
EAM	Electroanatomic mapping
fEGM	Fractionated electrograms
GP	Ganglionated plexus
HDFM	High-density fractionation mapping
HFS	High-frequency stimulation
HR	Heart rate
I^2^	I-squared statistic (heterogeneity)
LA	Left atrium
M	Mixed (type of syncope)
POTS	Postural orthostatic tachycardia syndrome
PC	Prospective cohort
PRISMA	Preferred reporting items for systematic reviews and meta-analyses
QM	Q-test for moderators
RCT	Randomised controlled trial
RA	Right atrium
RFA	Radiofrequency ablation
R	Recurrent (type of syncope)
REML	Restricted maximum likelihood
SA	Spectral analysis
SDNN	Standard deviation of normal-to-normal intervals
V	Vasodepressor (type of syncope)
VVS	Vasovagal syncope
