# Supplementary material for: Cardioneuroablation for Vasovagal Syncope: An Updated Systematic Review and Single-Arm Meta-Analysis
Source: Biomedicines. 2025 Jul 18;13(7):1758. doi: 10.3390/biomedicines13071758 (PMC12292116; doi:10.3390/biomedicines13071758)
Supplement: Supplementary file 1 [file biomedicines-13-01758-s001.zip › biomedicines-3729075-supplementary.pdf]

Supplementary Figures

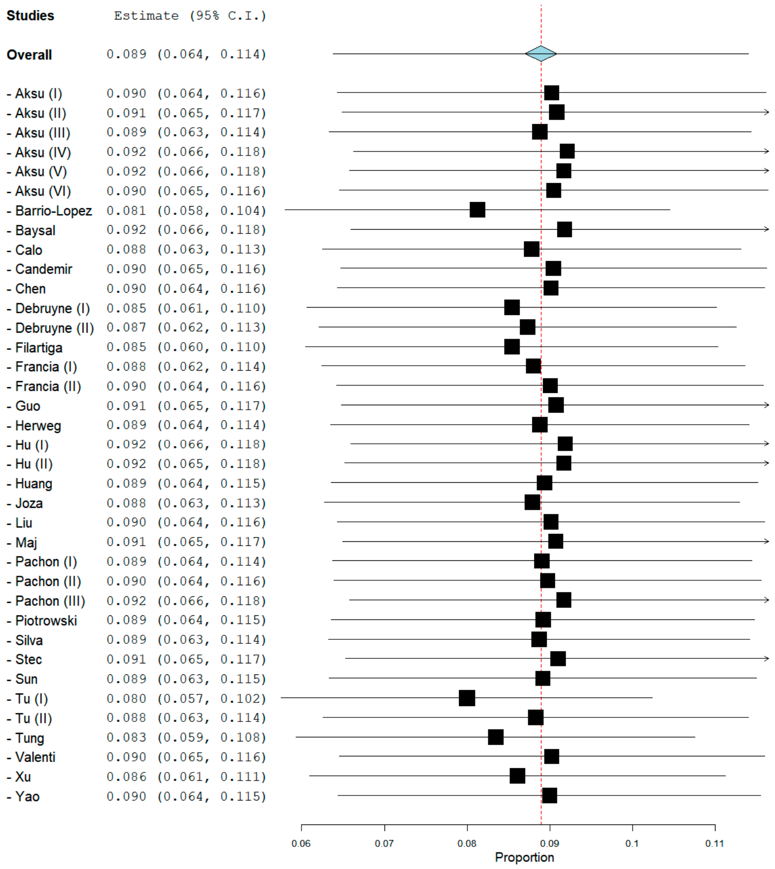

Figure S1. Leave-one-out Forest Plot of VVS recurrence after cardioneuroablation [18–54].

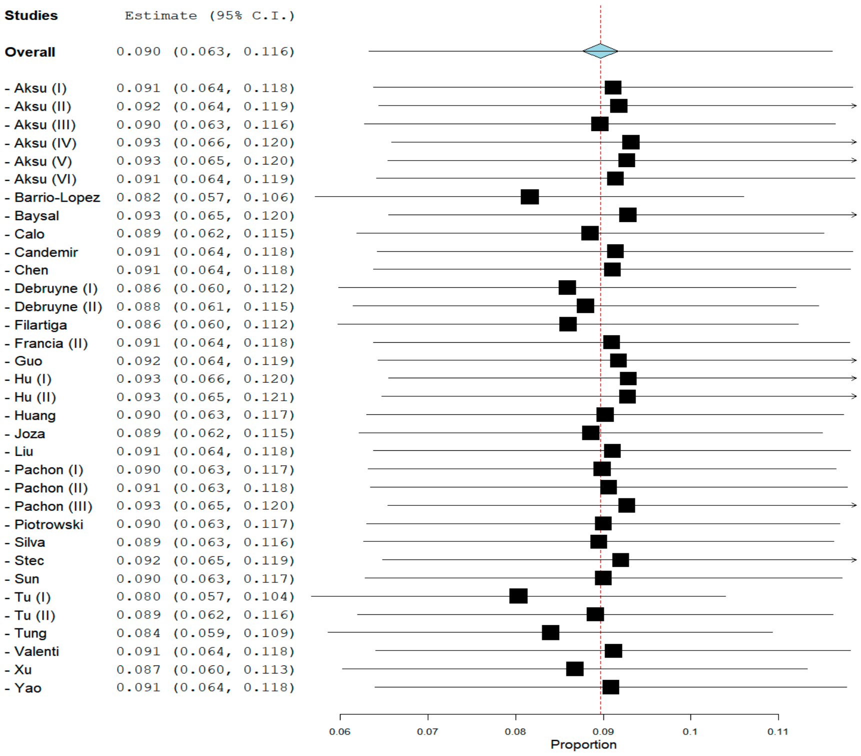

Figure S2. Leave-one-out Forest Plot on RFA subgroup [18–31,33,34,36–40,42–54].

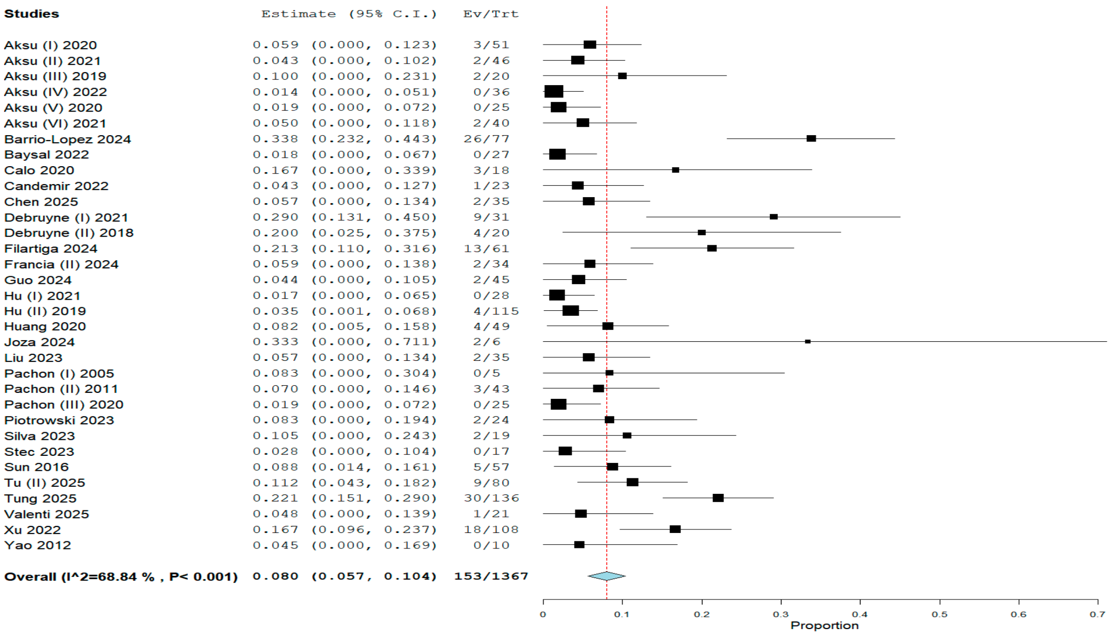

Figure S3. Forest Plot on RFA subgroup without Tu (I)[49].

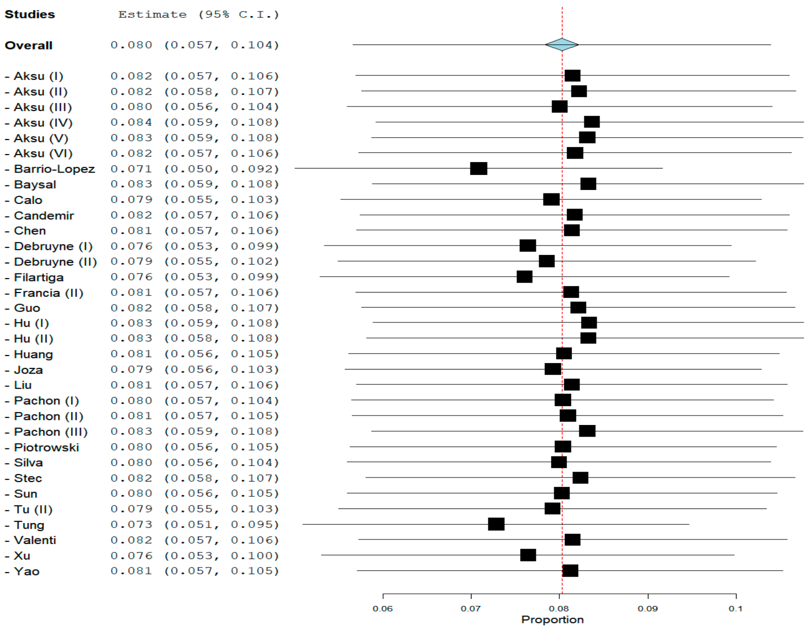

Figure S4. Leave-one-out Forest Plot on RFA subgroup without Tu (I)[49] and Barrio-Lopez[24] studies.

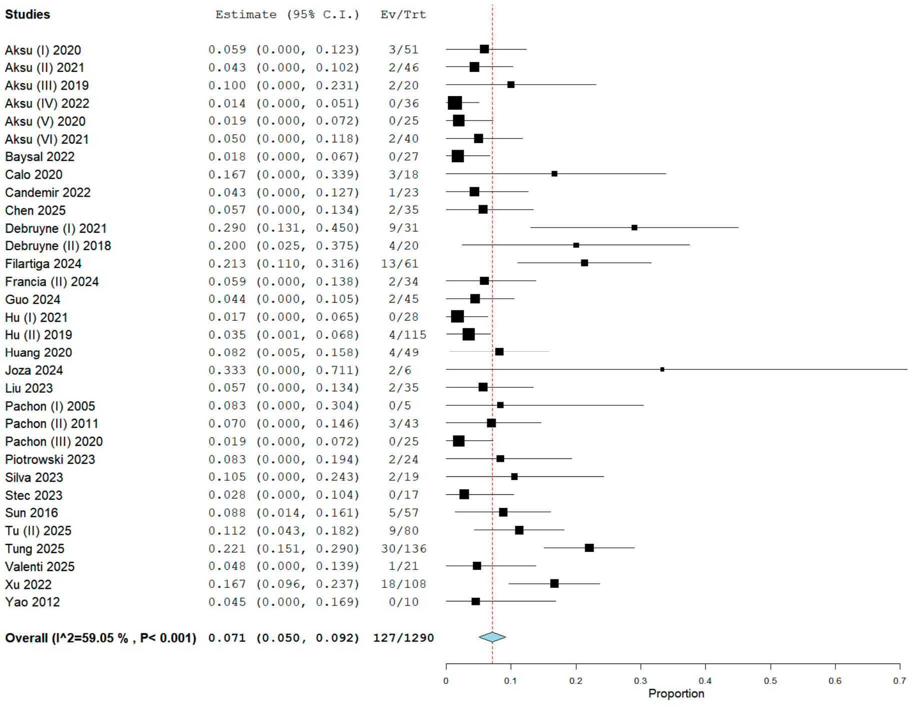

Figure S5. Forest Plot on RFA subgroup without Tu (I)[49] and Barrio-Lopez[24] studies.

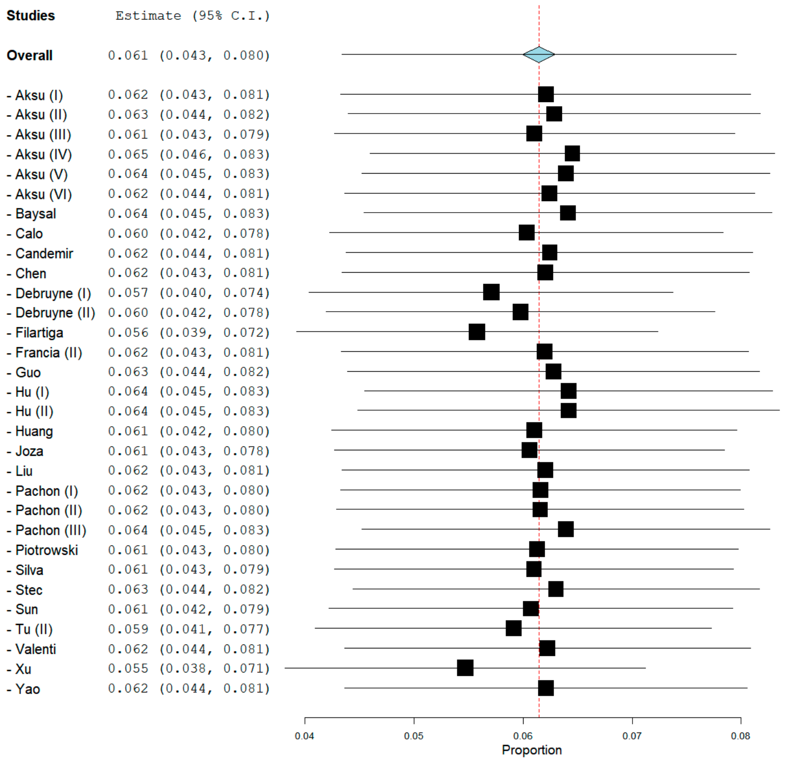

Figure S6. Leave-one-out Forest Plot on RFA subgroup without Tu (I)[49], Barrio-Lopez[24] and Tung[51] studies.

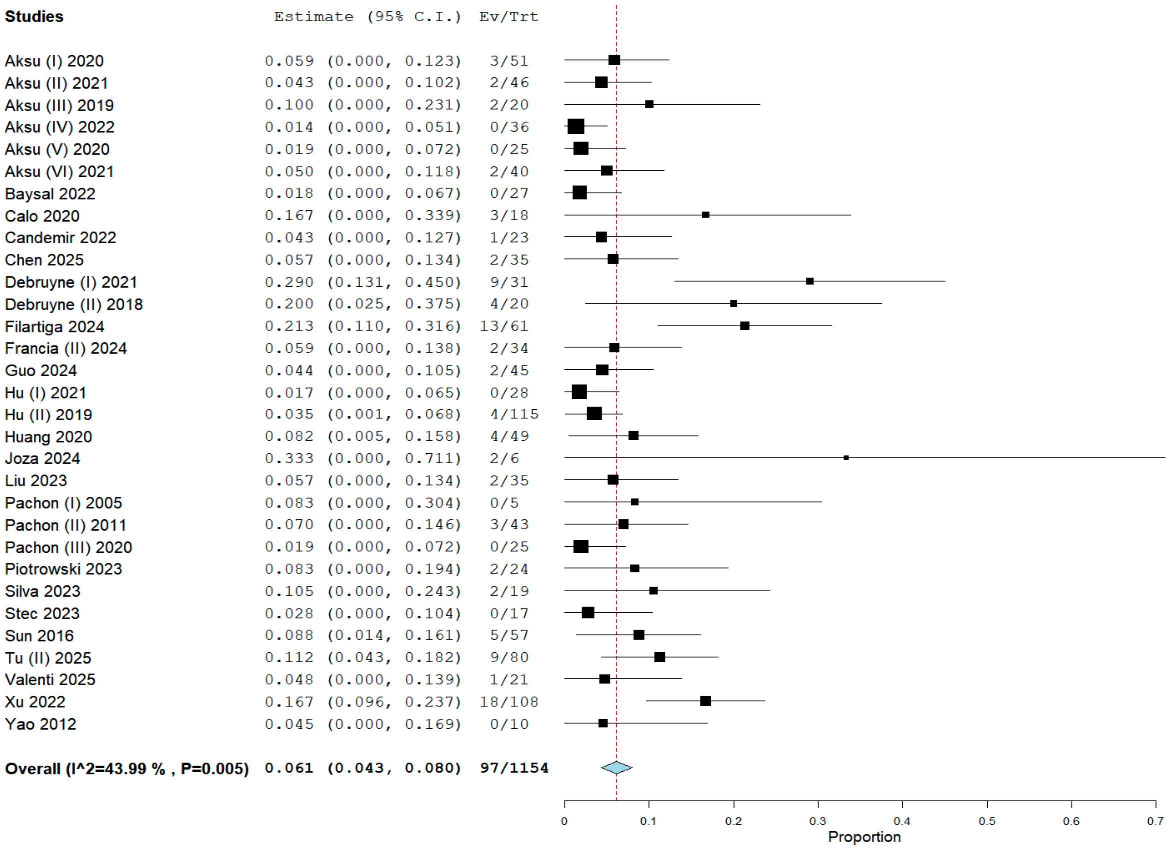

Figure S7. Forest Plot on RFA subgroup without Tu (I)[49], Barrio-Lopez[24] and Tung[51] studies.

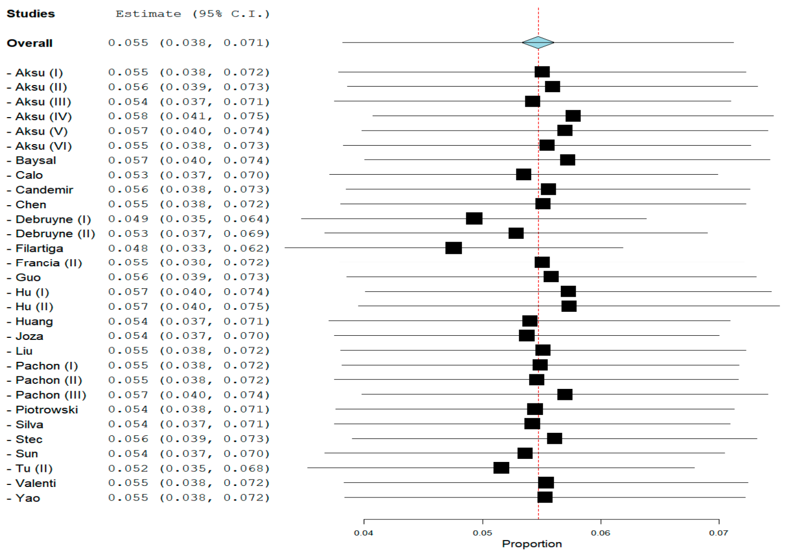

Figure S8. Leave-one-out Forest Plot on RFA subgroup without Tu (I)[49], Barrio-Lopez[24], Tung[51] and Xu[53] studies.

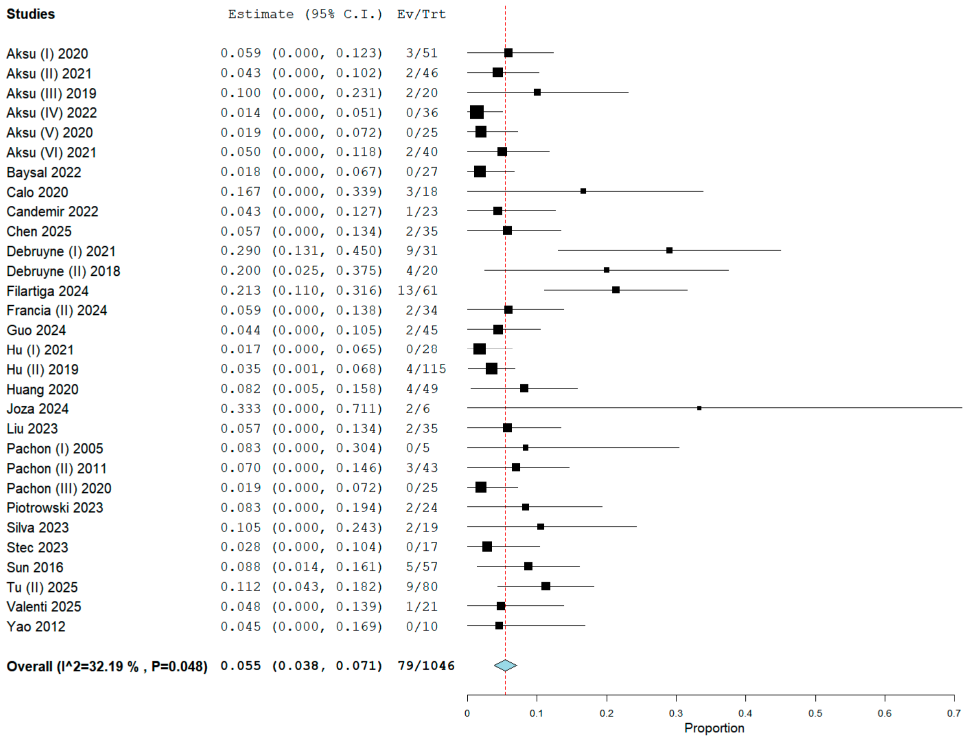

Figure S9. Forest Plot on RFA subgroup without Tu (I)[49], Barrio-Lopez[24], Tung[51] and Xu[53] studies.

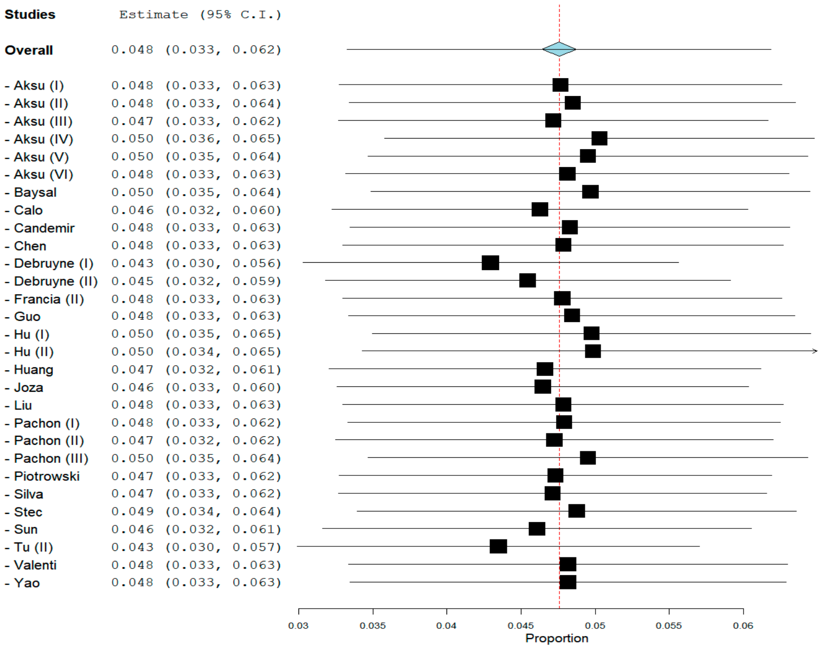

Figure S10. Leave-one-out Forest Plot on RFA subgroup without Tu (I)[49], Barrio-Lopez[24], Tung[51], Xu[53] and Filartiga[31] studies.

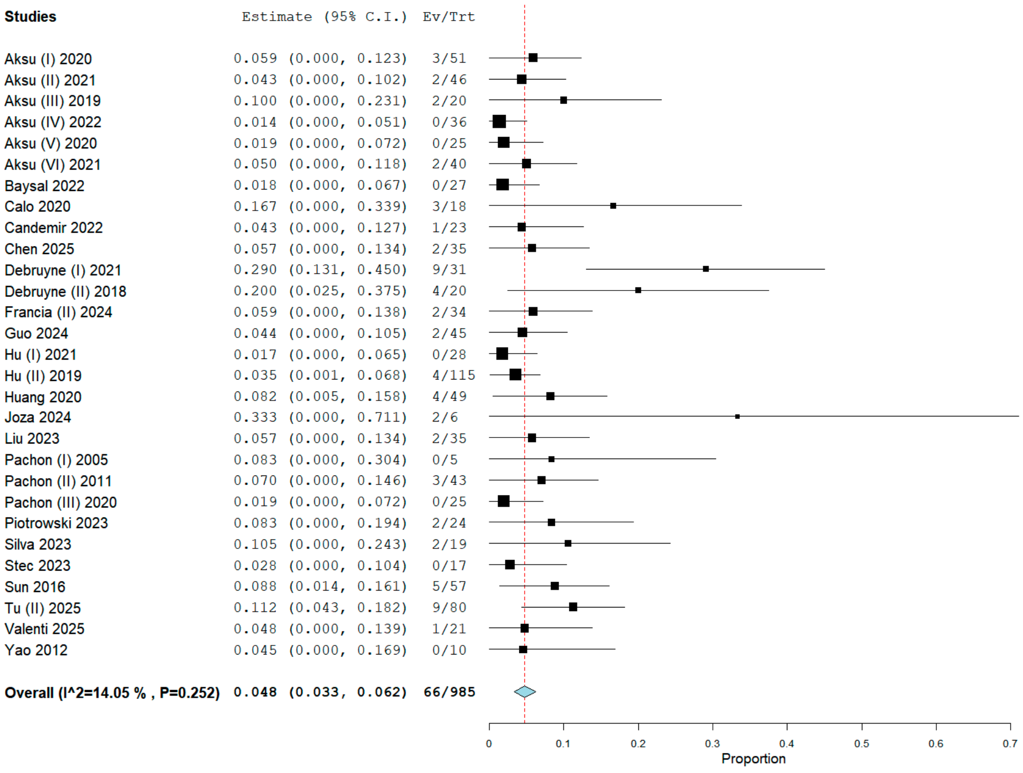

Figure S11. Forest Plot on RFA subgroup without Tu (I)[49], Barrio-Lopez[24], Tung[51], Xu[53] and Filartiga[31] studies.

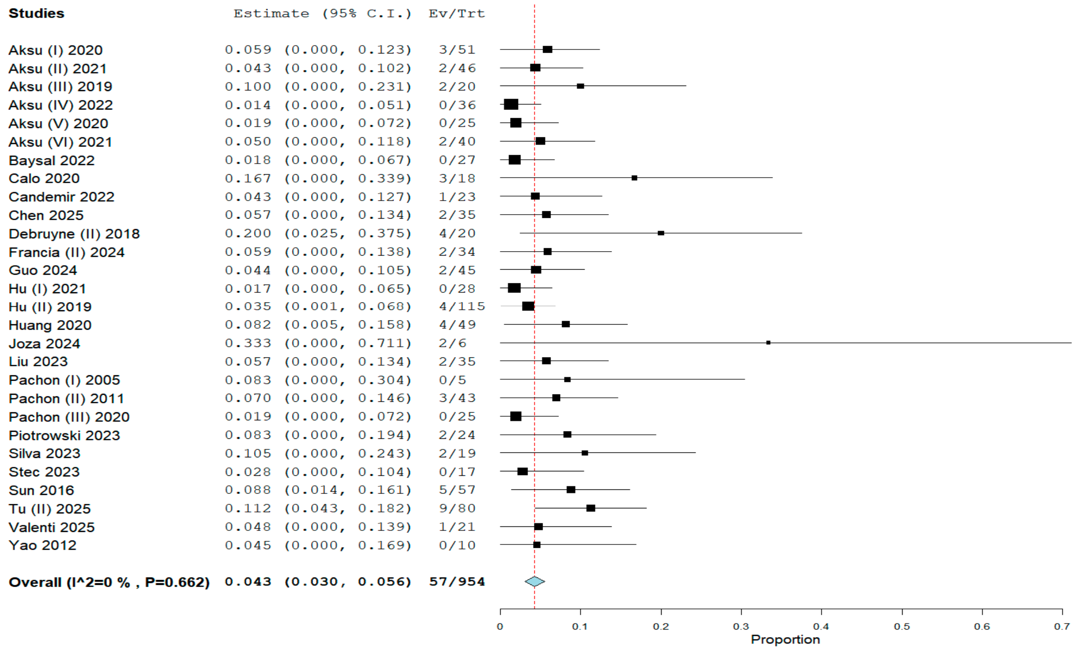

Figure S12. Forest Plot for the RFA subgroup without Tu (I)[49], Barrio-Lopez[24], Tung[51], Xu[53], Filartiga[31] and Debruyne (I)[29] studies.

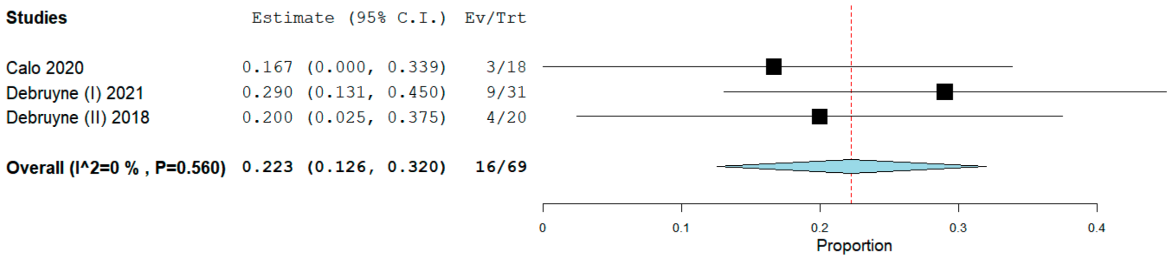

Figure S13. Forest Plot on RA localisation subgroup without Candemir[27].

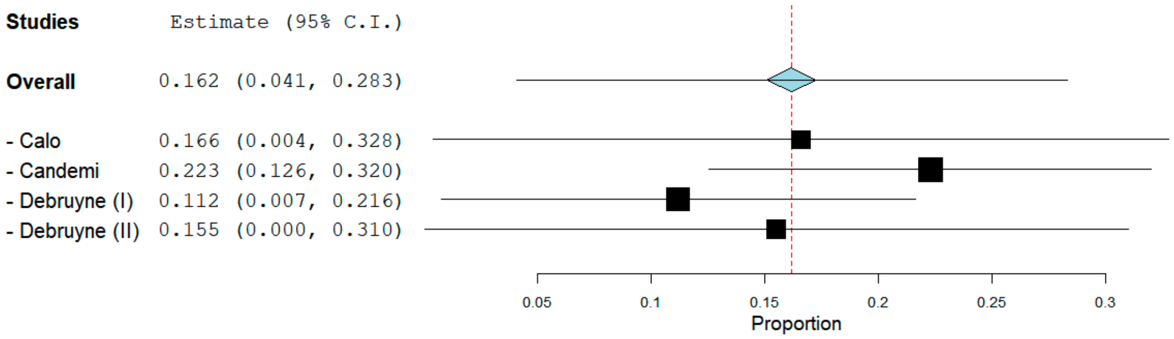

Figure S14. Leave-one-out Forest Plot on RA localisation subgroup [26,27,29,30].

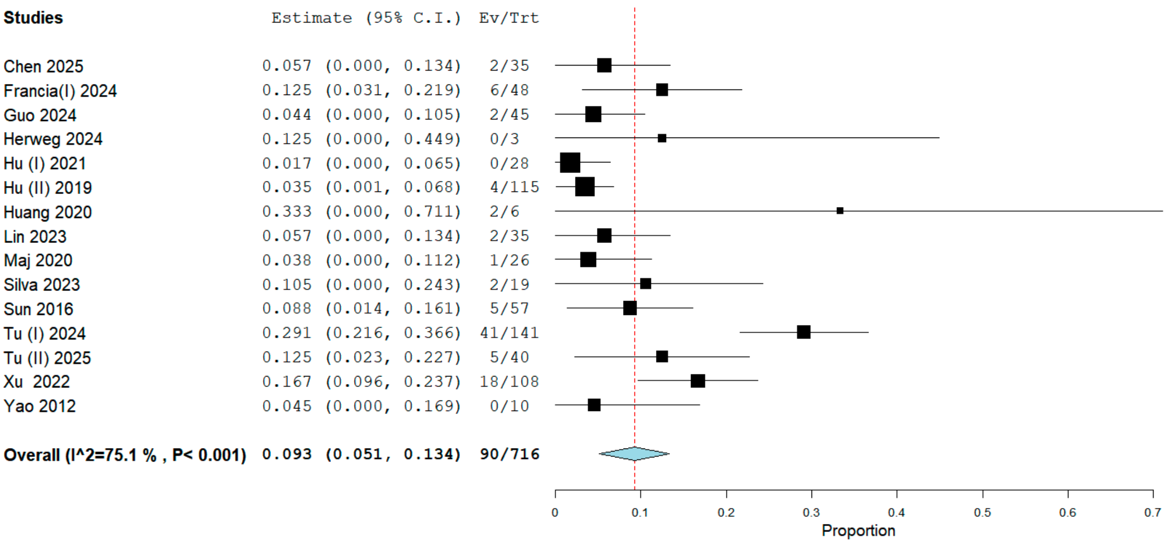

Figure S15. Forest Plot on LA localisation subgroup [28,32,34–38,40,41,46,47,50,54].

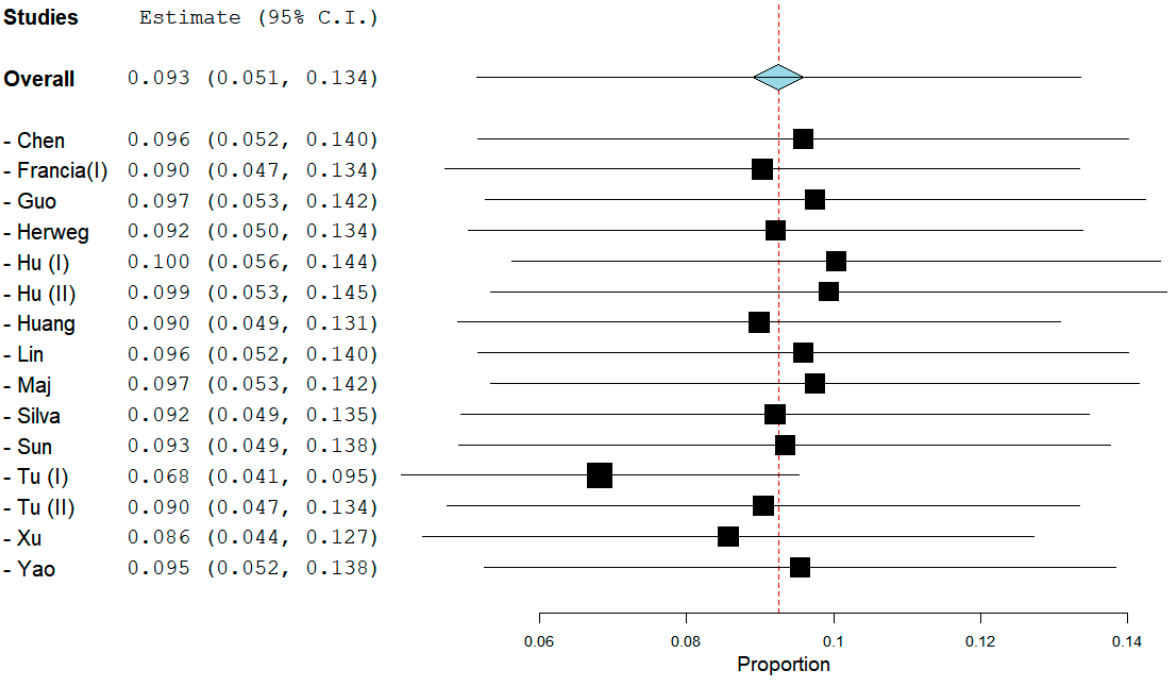

Figure S16. Leave-one-out Forrest Plot on LA localisation subgroup [28,32,34–38,40,41,46,47,50,54].

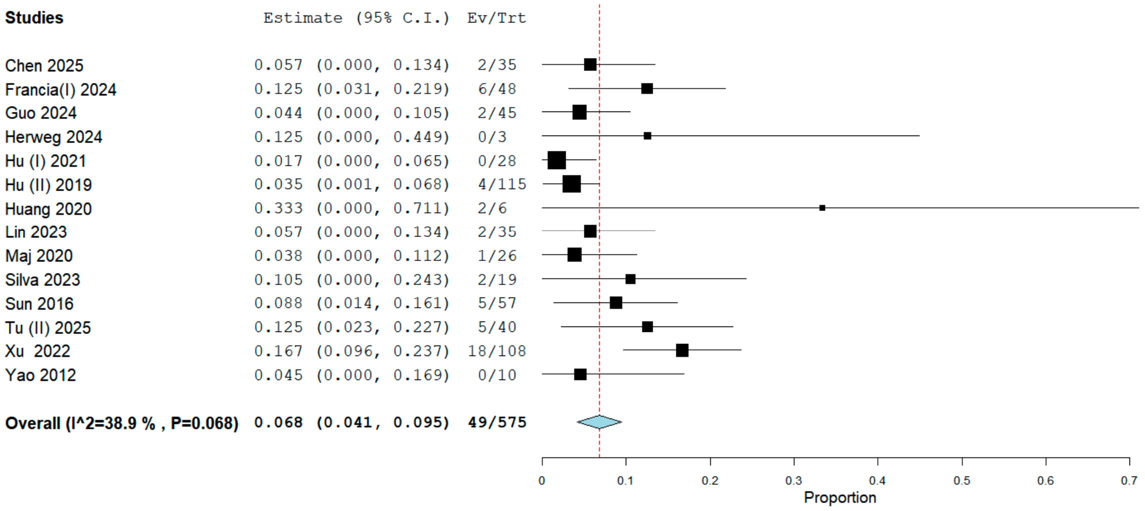

Figure S17. Forest Plot on LA localisation subgroup without Tu (I)[49].

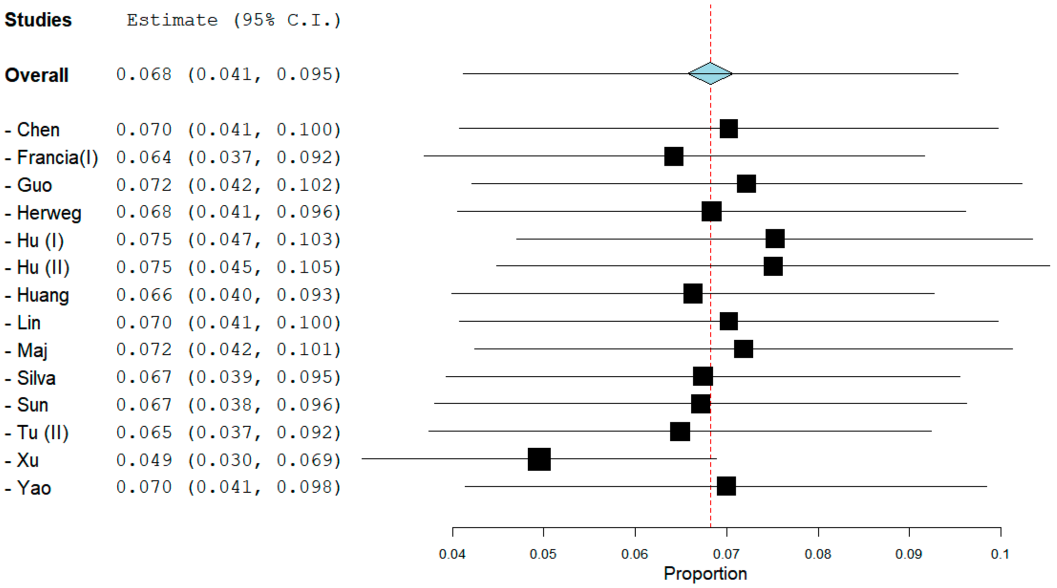

Figure S18. Leave-one-out Forrest Plot on LA localisation subgroup without Tu (I)[49].

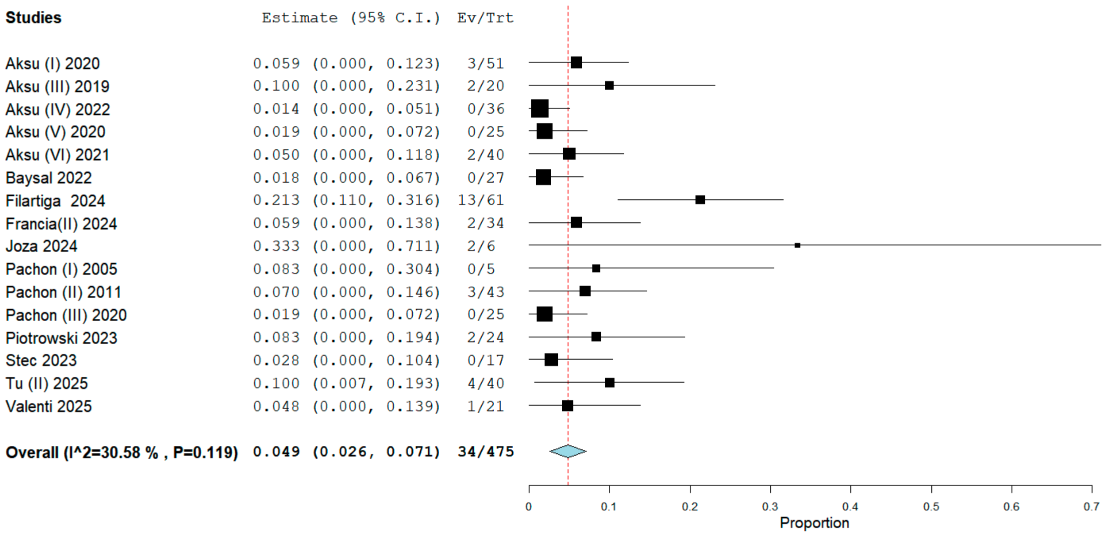

Figure S19. Forest Plot on Biatrial localisation subgroup [18,20–23,25,32,39,42–45,48,50,52].

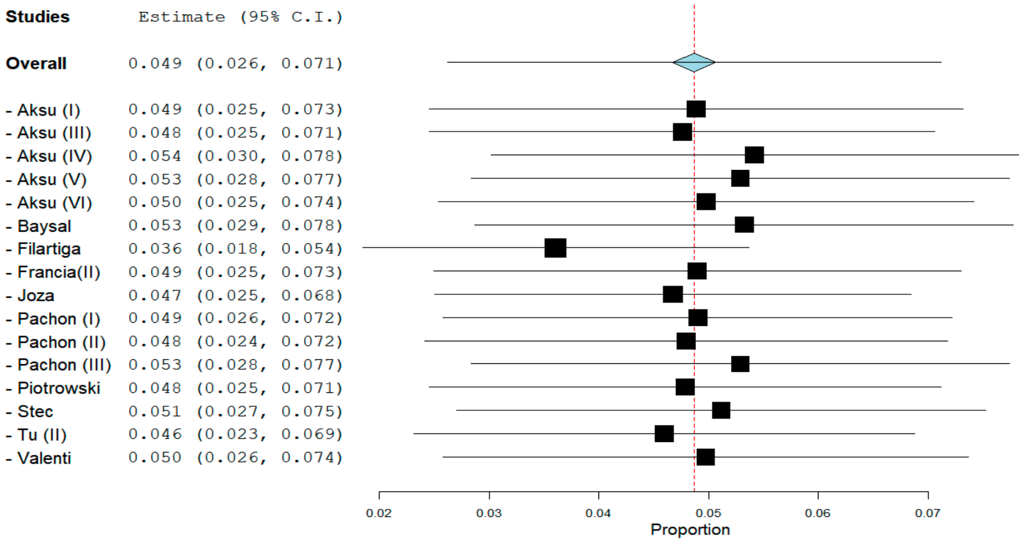

Figure S20. Leave-one-out Forrest Plot on Biatrial localisation subgroup [18,20–23,25,32,39,42–45,48,50,52].

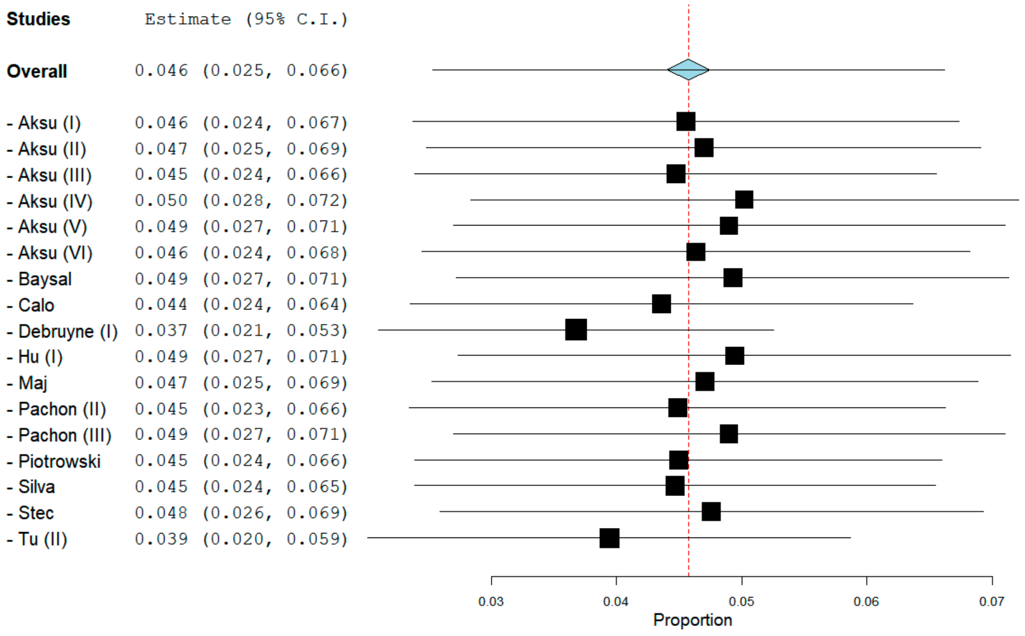

Figure S21. Leave-one-out Forrest Plot of VVS recurrence on 30 – 39.9 years mean age subgroup [18–23,25,26,36,41,43–45,47,48,50].

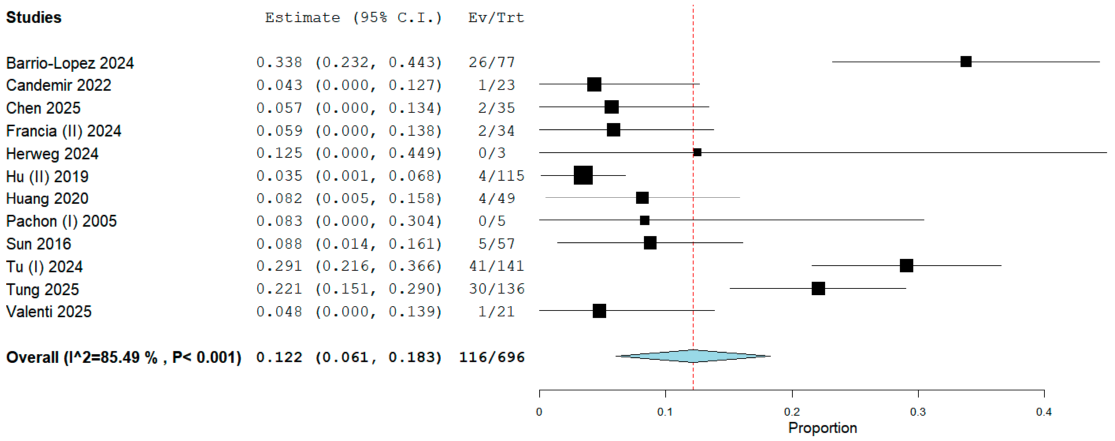

Figure S22. Forrest Plot of VVS Recurrence on 40 – 49.9 years mean age subgroup [24,27,28,32,35,37,42,46,49,51,52].

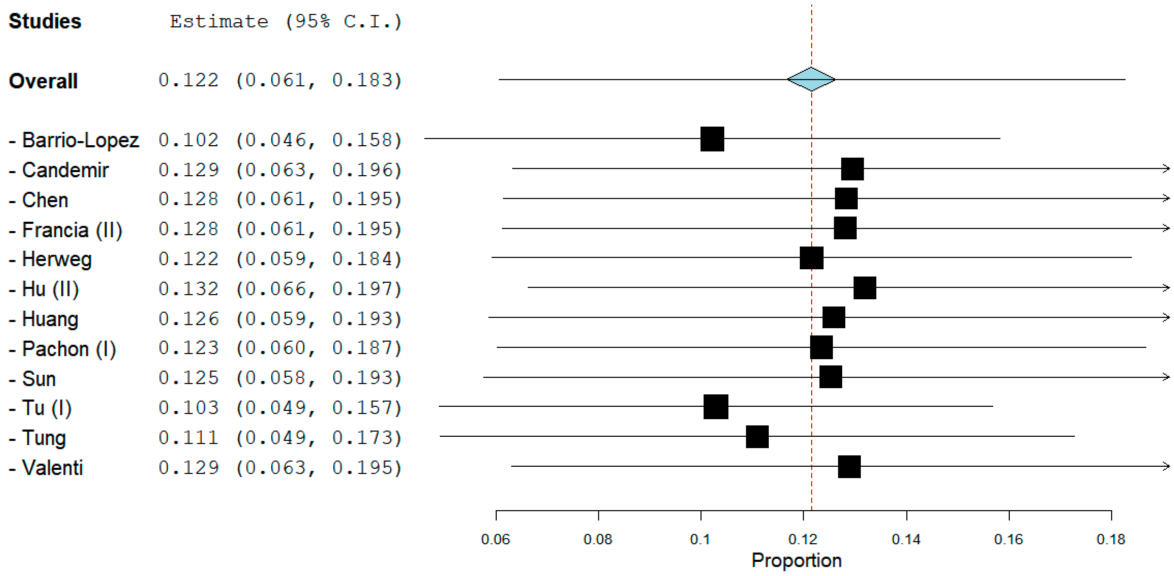

Figure S23. Leave-one-out Forest Plot of VVS Recurrence on 40 – 49.9 years mean age subgroup [24,27,28,32,35,37,42,46,49,51,52].

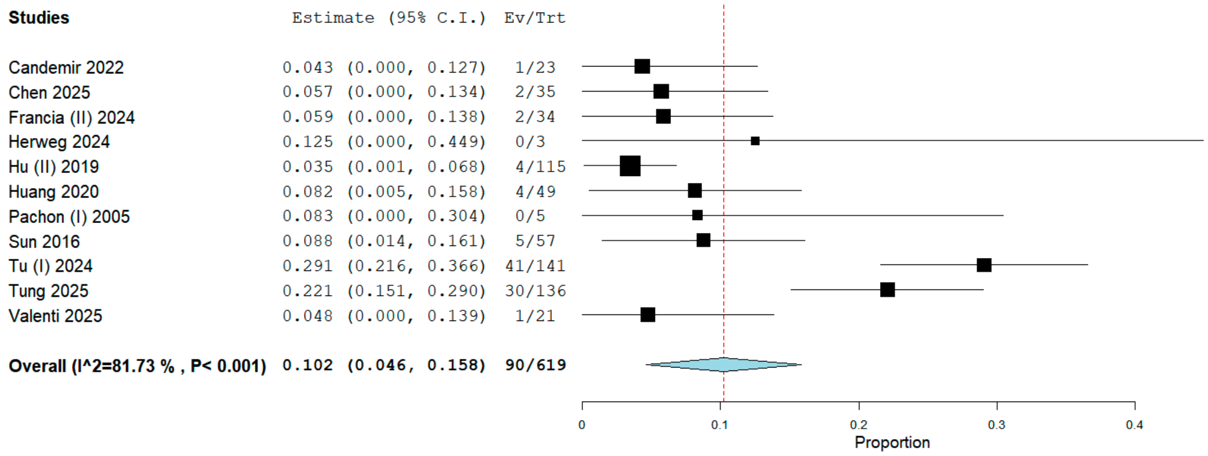

Figure S24. Forest Plot of VVS Recurrence on 40 – 49.9 years mean age subgroup without Barrio-Lopez [24].

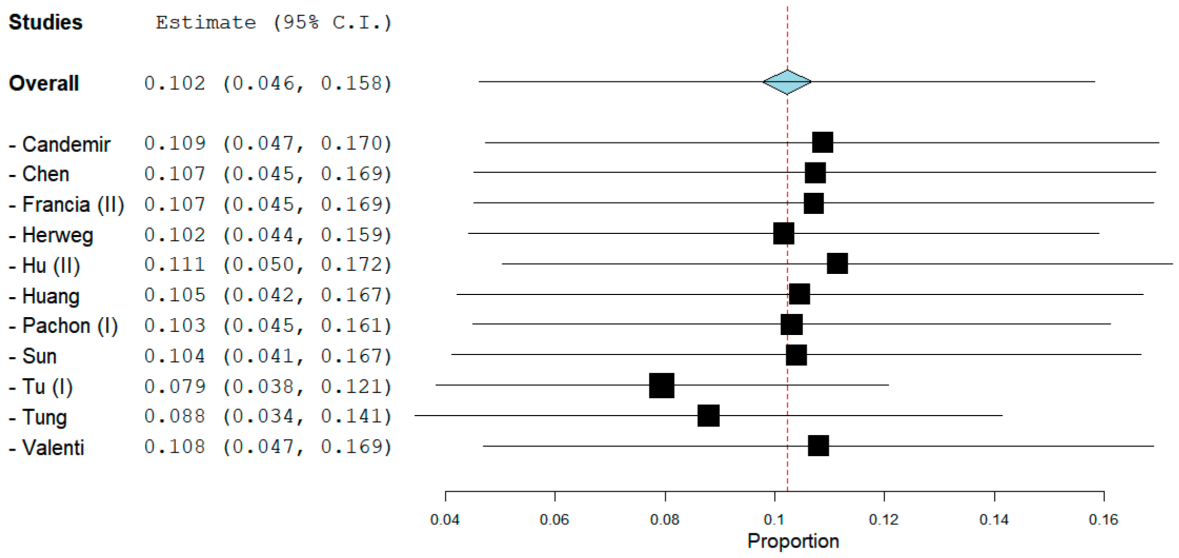

Figure S25. Leave-one-out Forest Plot of VVS Recurrence on 40 – 49.9 years mean age subgroup without Barrio-Lopez[24].

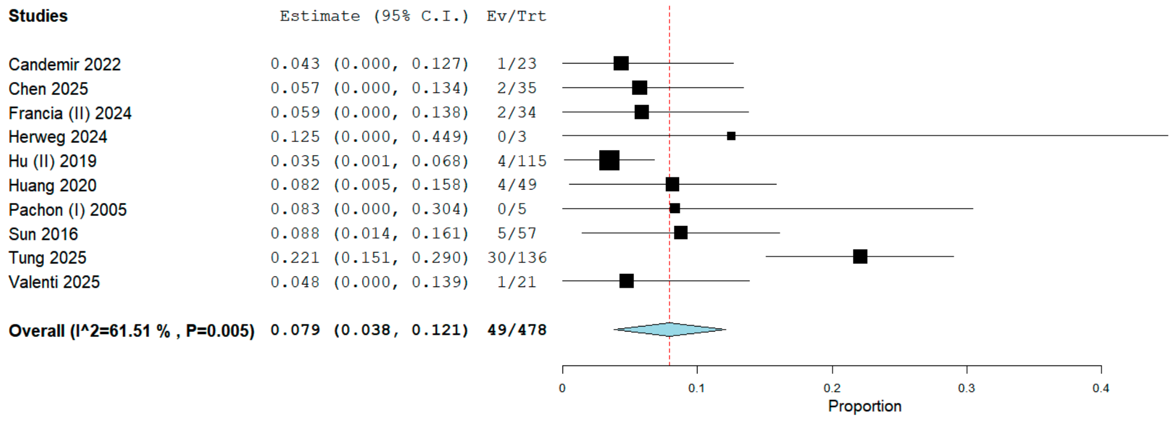

Figure S26. Forest Plot of VVS Recurrence on 40 – 49.9 years mean age subgroup without Barrio-Lopez[24] and Tu(I)[49].

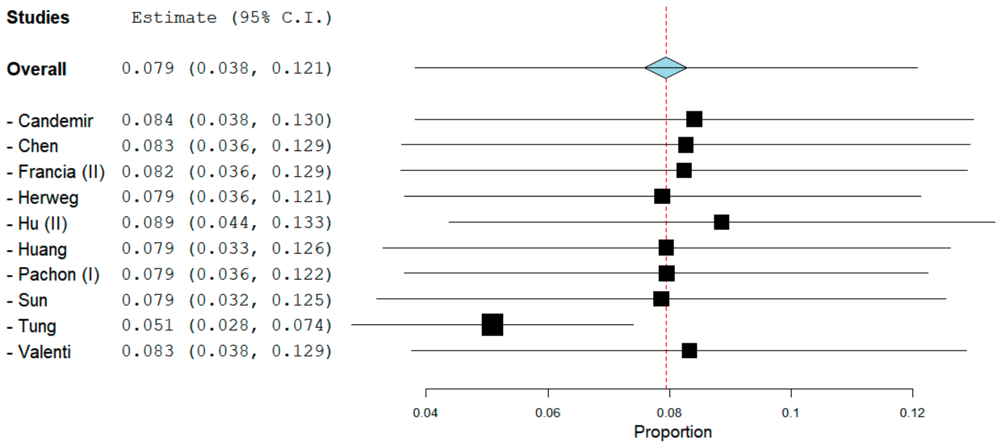

Figure S27. Leave-one-out Forest Plot of VVS Recurrence on 40 – 49.9 years mean age subgroup without Barrio-Lopez[24] and Tu(I)[49].

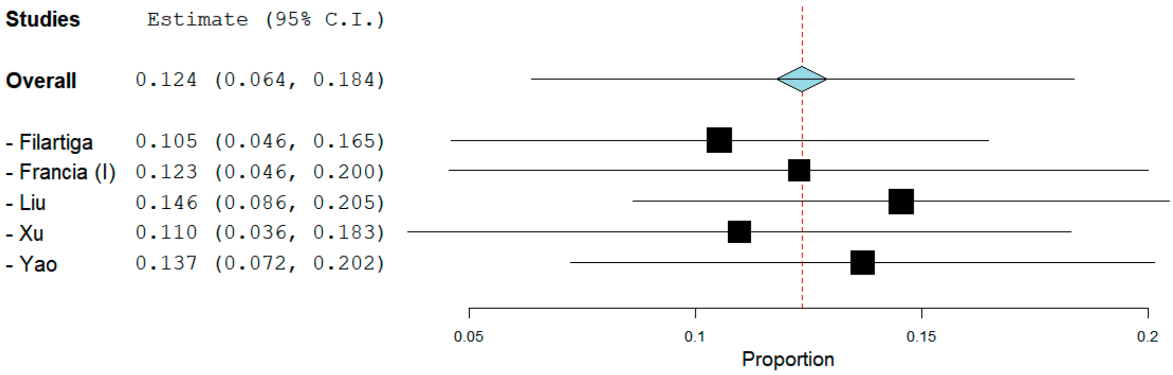

Figure S28. Leave-one-out Forest Plot of VVS Recurrence > 50 years mean age subgroup [31,32,40,53,54].

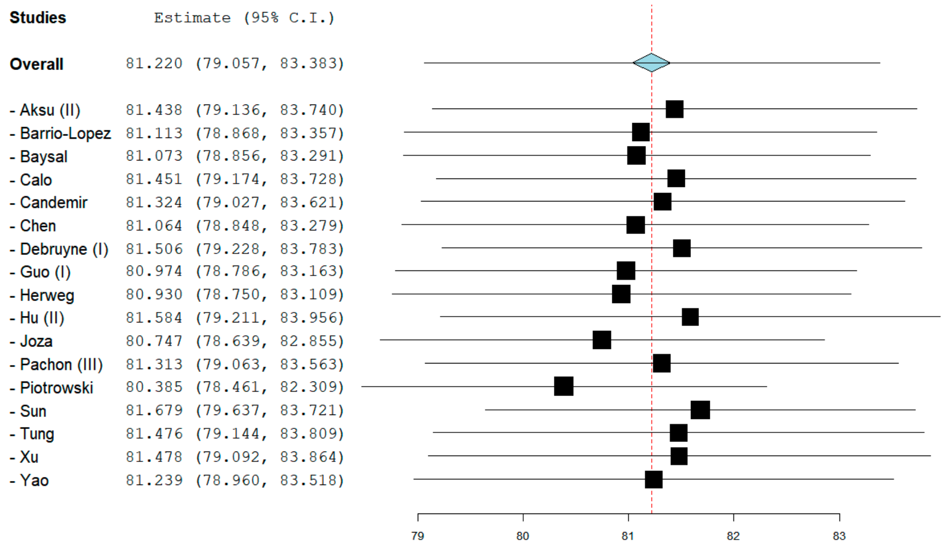

Figure S29. Leave-one-out Forest Plot on Heart Rate after CNA [19, 24-29, 34-35, 37, 39, 44-46, 51, 53-54].

[19,24–29,34,35,37,39,44–46,51,53,54

Figure S30. Leave-one-out Forrest Plot on SDNN before CNA [[26–29,42,44–46,53,54].

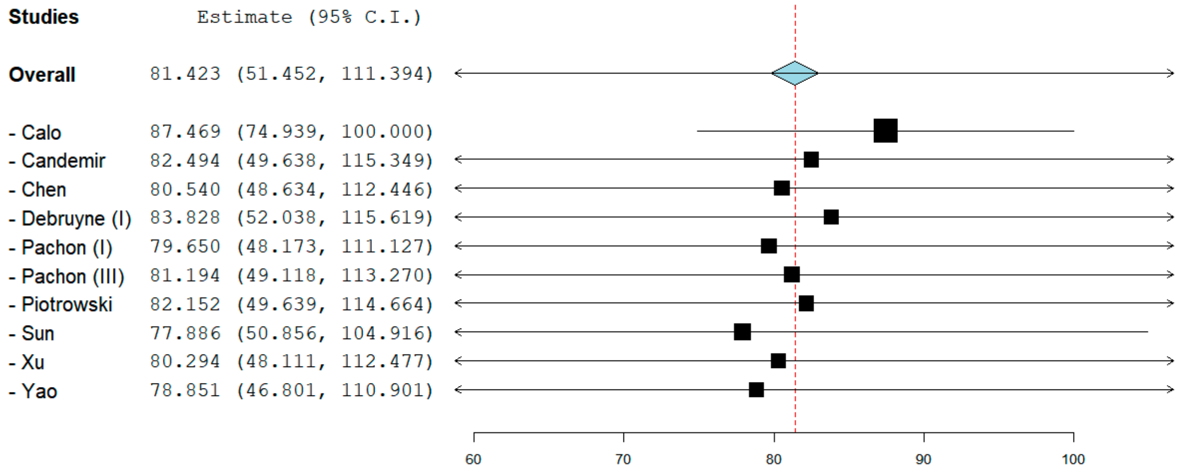

Figure S31. Leave-one-out Forest plot of SDNN after CNA [26–29,42,44–46,53,54].

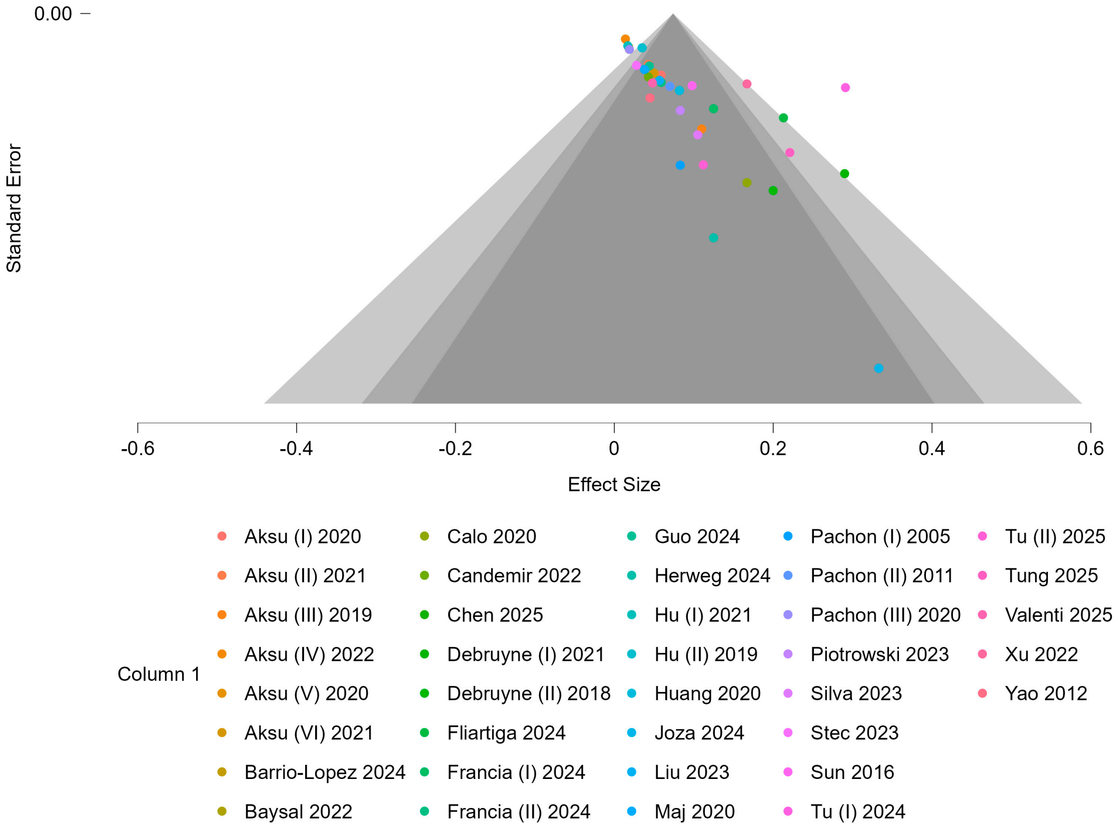

Figure S32. Funnel Plot of assessment potential publication bias [18-54].

[18–54

Funnel Plot Asymmetry Tests

Meta-Regression Test for Funnel Plot Asymmetry

	Asymmetry Test		Limit Estimate		
Estimates	z	p	Estimate	Lower 95% CI	Upper 95% CI
37	7.793	< .001	-0.022	-0.041	-0.003

Weighted Regression Test for Funnel Plot Asymmetry

	Asymmetry Test			Limit Estimate		
Estimates	t	df	p	Estimate	Lower 95% CI	Upper 95% CI
37	6.280	35	< .001	-0.022	-0.046	0.003

Rank Correlation Test for Funnel Plot Asymmetry

Estimates	τ	p
37	0.656	< .001

Figure S33. Funnel Plot Asymmetry Tests – statistical tests, Egger's and Begg's.

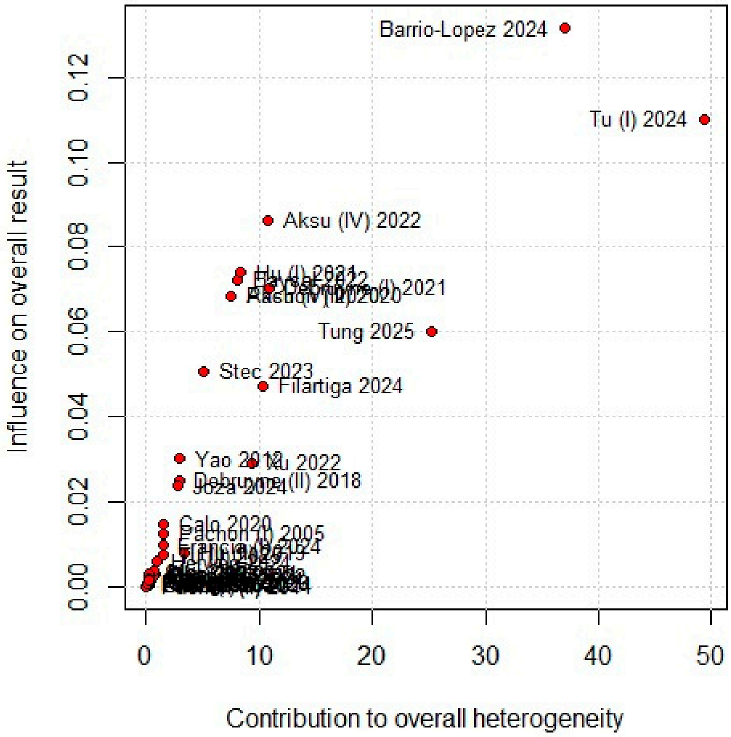

Figure S34. Baujat Plot highlighting studies that have a disproportionate impact on the overall effect size and contribute to between-study heterogeneity [18–54].
